# Exploring robust architectures for deep artificial neural networks

**DOI:** 10.1038/s44172-022-00043-2

**Published:** 2022-12-17

**Authors:** Asim Waqas, Hamza Farooq, Nidhal C. Bouaynaya, Ghulam Rasool

**Affiliations:** 1 Machine Learning Department, Moffitt Cancer Center, Tampa, FL USA; 2 Department of Radiology, University of Minnesota, Minneapolis, MN USA; 3 Department of Electrical and Computer Engineering, Rowan University, Glassboro, NJ USA

**Keywords:** Scientific community, Science, technology and society, Computer science, Computational science, Information technology

## Abstract

The architectures of deep artificial neural networks (DANNs) are routinely studied to improve their predictive performance. However, the relationship between the architecture of a DANN and its robustness to noise and adversarial attacks is less explored, especially in computer vision applications. Here we investigate the relationship between the robustness of DANNs in a vision task and their underlying graph architectures or structures. First we explored the design space of architectures of DANNs using graph-theoretic robustness measures and transformed the graphs to DANN architectures using various image classification tasks. Then we explored the relationship between the robustness of trained DANNs against noise and adversarial attacks and their underlying architectures. We show that robustness performance of DANNs can be quantified before training using graph structural properties such as topological entropy and Olivier-Ricci curvature, with the greatest reliability for complex tasks and large DANNs. Our results can also be applied for tasks other than computer vision such as natural language processing and recommender systems.

## Abstract (web summary)

Asim Waqas and colleagues investigated the relationship between the architectures of deep artificial neural networks (DANNs) and their robustness to noise and adversarial attacks in computer vision. The researchers found that the robustness of DANNs was highly correlated with graph-theoretic measures of entropy and curvature. This finding could help design more robust DANN architectures.

## Introduction

The architecture or structure of a deep artificial neural network (DANN) is defined by the connectivity patterns among its constituent artificial neurons. The mere presence or absence of a connection between two neurons or a set of neurons may provide a useful prior and improve the predictive performance of a DANN. A range of architectures has been developed over years to tackle various machine learning tasks in computer vision, natural language processing, and reinforcement learning^1–5^. In general, the process of the development of DANN architectures is manual, iterative, and time consuming. AutoML and neural architecture search (NAS) attempt to use machine learning and search the design space of DANNs for architectures that may yield maximum test data accuracy. After the selection of a suitable DANN architecture for the given task, the optimal values of the connections (parameters or weights) are found using the training dataset and the well-known gradient descent or one of its variant algorithms^6,7^. Recently, considerable research efforts have been focused on automating the laborious task of DANN architecture design and development using techniques of autoML and NAS^8–11^. However, all such efforts are primarily focused on improving the test accuracy of the DANN on the given task. In the real world, DANNs face the challenging problem of maintaining their predictive performance in the face of uncertainties and noise in the input data^13,14^. The noise can be in the attributes of the input samples (attribute noise), and it can be in the associated class label (label noise). DANNs exhibit intrinsic robustness to label noise, and the test accuracy of DANNs drop only marginally against the tens of percentage increase in the label noise^15–17^. So, the term “noise” in this work refers to the more hostile sample noise, called the attribute noise. The challenge of noise in the attributes of data is further exacerbated for mission-critical application areas, such as clinical diagnosis, autonomous driving, financial decision-making, cyberspace, and defense. Although we have used computer vision for hypothesis testing in this work, we believe that the proposed concepts are equally applicable to other fields mentioned above. Ideally, a real world deployment-ready DANN should be robust to or equivalently maintain its predictive performance against two different types of noise, natural and malicious. The natural noise is related to the out-of-distribution generalization. Such noise is caused by the day-to-day changes in input data, e.g., changes in the hardware or software configurations used for processing input data. The malicious or adversarial noise is generated by an adversary for fooling the DANN into producing an erroneous decision^18^. The adversarial attacks can be at training time (poisoning attack) or at inference time (evasion attack)^19^. The attacks themselves can be targeted in the feature-space^18^ or in the problem space^20^. The attacker can have a perfect knowledge (white-box attacks), limited knowledge (gray-box attacks), or zero knowledge (black-box attacks)^20^. Here, the knowledge *θ* = (*D*, *X*, *f*, *w*) is a set that may contain training data *D*, features *X*, model *f,* and its parameters *w*. Black-box attacks are strict subset of white-box attacks, and white-box attacks perform better than other attacks against a DANN^21^. This means that gradient-based attacks outperform gradient-free attacks^21^. Following these facts and the Kerckhoffs’ principle^22^, in this work, we have employed white-box attacks assuming attacker’s perfect knowledge at inference time. Moreover, this work focuses on evaluating the inherent robustness of DANNs to identify the architectures that have a natural relative immunity to adversaries and insults. Mechanisms on improving the robustness of DANNs is not covered in this work.

It has been shown with the help of Percolation theory that the architecture or structure underlying a network of any real-world system may play a key role in defining its robustness to various insults and attacks^23^. Graph-theoretic measures, such as network topological entropy and Ollivier-Ricci curvature, successfully quantify the functional robustness of various networks^24^. Examples include studying the behavior of cancer cells, analyzing the fragility of financial networks, studying the robustness of brain networks, tracking changes attributable to age and autism spectrum disorder (ASD), and explaining cognitive impairment in Multiple Sclerosis (MS) patients^25–28^. Recently, the relationship between the architectures of DANNs (quantified by various graph-theoretic measures before training) and their predictive accuracy (available after training) has been established^8,9^. Various graph-theoretic measures (e.g., path length and clustering coefficient) calculated from the architectures of DANNs are quantitatively linked to their accuracy on various image classification tasks. However, the relationship between the graph-theoretic measures related to the robustness (e.g., entropy and Ollivier-Ricci curvature) of the architecture of DANNs and their performance against natural and adversarial noise has never been explored. Establishing such a relationship will allow the autoML and NAS research community to design and develop robust DANNs without training and testing these architectures.

Graph-theoretic measures that are related to the vulnerability and robustness of networks can be categorized into graph connectivity measures, adjacency spectrum measures, and Laplacian spectrum measures^29^. Based on the graph properties such as Ollivier-Ricci curvature, betweenness centrality, and shortest path length between nodes, more advanced network measures have been recently proposed. For example, graph and node-based multifractal analysis^30,31^, and fitness-based network efficiency for heterogeneous nodes^32^ quantify the topology and robustness of complex networks. In this work, we study graph-theoretic properties of the architectures of DANNs to quantify their test-time robustness. Specifically, we use the graph measures of topological entropy and curvature of the architecture of DANNs as robustness metrics. We have considered all three aforementioned categories of graph-robustness measures in our experiments. Our choice of reporting the curvature and entropy as the robustness measures of DANNs is based on empirical evidence presented in this paper. We make two distinct research contributions to the robustness analysis of DANNs: (1) We establish a quantitative relationship between the graph-theoretic robustness measures of entropy and curvature of DANNs (available before training) and the robustness of these DANNs to natural and adversarial noise (evaluated after training DANNs). Previous studies explored graph measures that relate to the performance of DANNs, but the robustness of DANNs through graph-robustness measures has never been studied. We show that graph entropy and curvature are related to DANNs’ robustness, and these structural measures can identify robust architectures of DANNs even before training for the given task. (2) We show that the relationship between the graph robustness measured using entropy and Ollivier-Ricci curvature and the robustness performance of DANN against noise and adversarial attacks becomes significantly stronger for complex tasks, larger datasets, and bigger DANNs. Given that the sizes of DANNs and the complexity of tasks/datasets are growing significantly for many real-world applications, the strong entropy-robustness relationship assumes greater importance. The autoML/NAS design problems where the robustness of DANNs is vital, our analysis can help identify robust architectures without the need to train and test these DANNs under various noisy conditions.

**Fig. 1:**
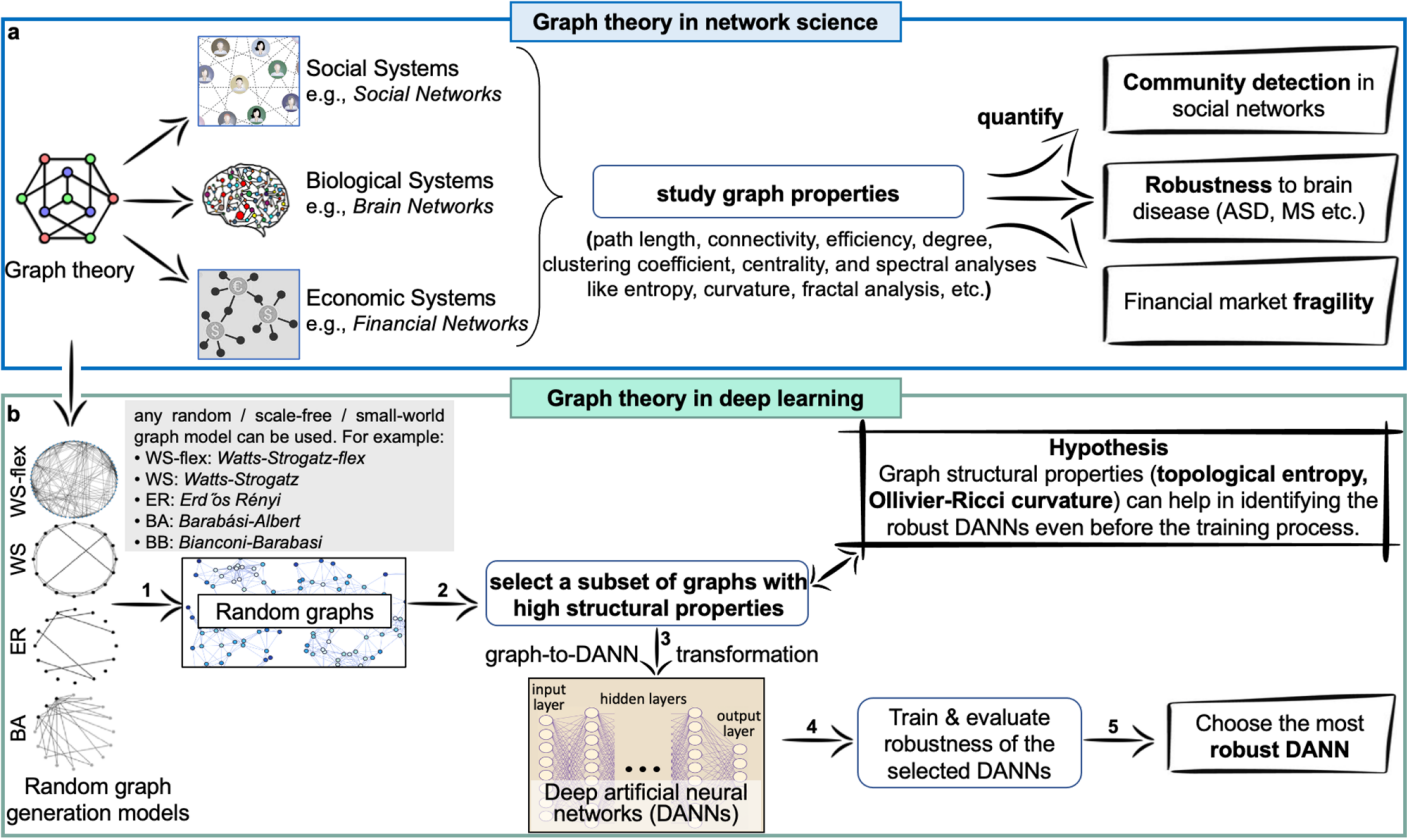
Exploring robustness of Deep Artificial Neural Networks (DANNs) with graph-theoretic measures. **a** In network science (NetSci), real-world systems such as brain networks, financial networks, and social networks are studied using graph-theoretic measures to quantify their robustness and fragility. **b** We use graph-theoretic measures established in NetSci to study graphs of architectures of DANNs. Using graph-theoretic robustness measures, we can find robust architectures for DANNs without exhaustively training and evaluating many DANNs.

In Fig. 1, we provide an overview of the proposed approach. Figure 1a illustrates how graph-theoretic measures are often applied in Network Science (NetSci) to study various real-world networks. The illustrated examples include biological systems, such as brain networks, economic systems, such as financial networks, and social systems such as social networks. Path length, graph connectivity, efficiency, degree measures, clustering coefficient, centrality, spectral measures (curvature, entropy), and fractal analysis are the graph-theoretic measures that researchers have employed for studying real-world networks^25–28,33,34^.

Figure 1b illustrates our proposed methodology. Our approach consists of five steps: (1) build random, scale-free, or small-world networks or graphs using classical families of graphs, (2) calculate graph-theoretic measures of these random graphs in the graph domain and select a small subset from the entire design space for further analysis, (3) convert selected random graphs into architectures of DANNs (e.g., MLPs, CNNs, ResNets, EfficientNets), (4) train, validate and test these DANNs under different natural noise and adversarial conditions, and (5) analyze and link the robustness of architectures (measured with graph-theoretic properties) to the performance of trained DANNs against natural noise and adversarial attacks. We hypothesize that the graph-theoretic measures that quantify the robustness of networks/graphs in the NetSci domain will also provide insight into the robustness of DANNs in the deep learning domain. We provide empirical evidence to support our hypothesis. We use the term DANN for deep artificial neural networks, graphs for unweighted directed acyclic graphs, and network for various networks as used in the network science (NetSci) domain.

## Results

### Graph design space

**Fig. 2:**
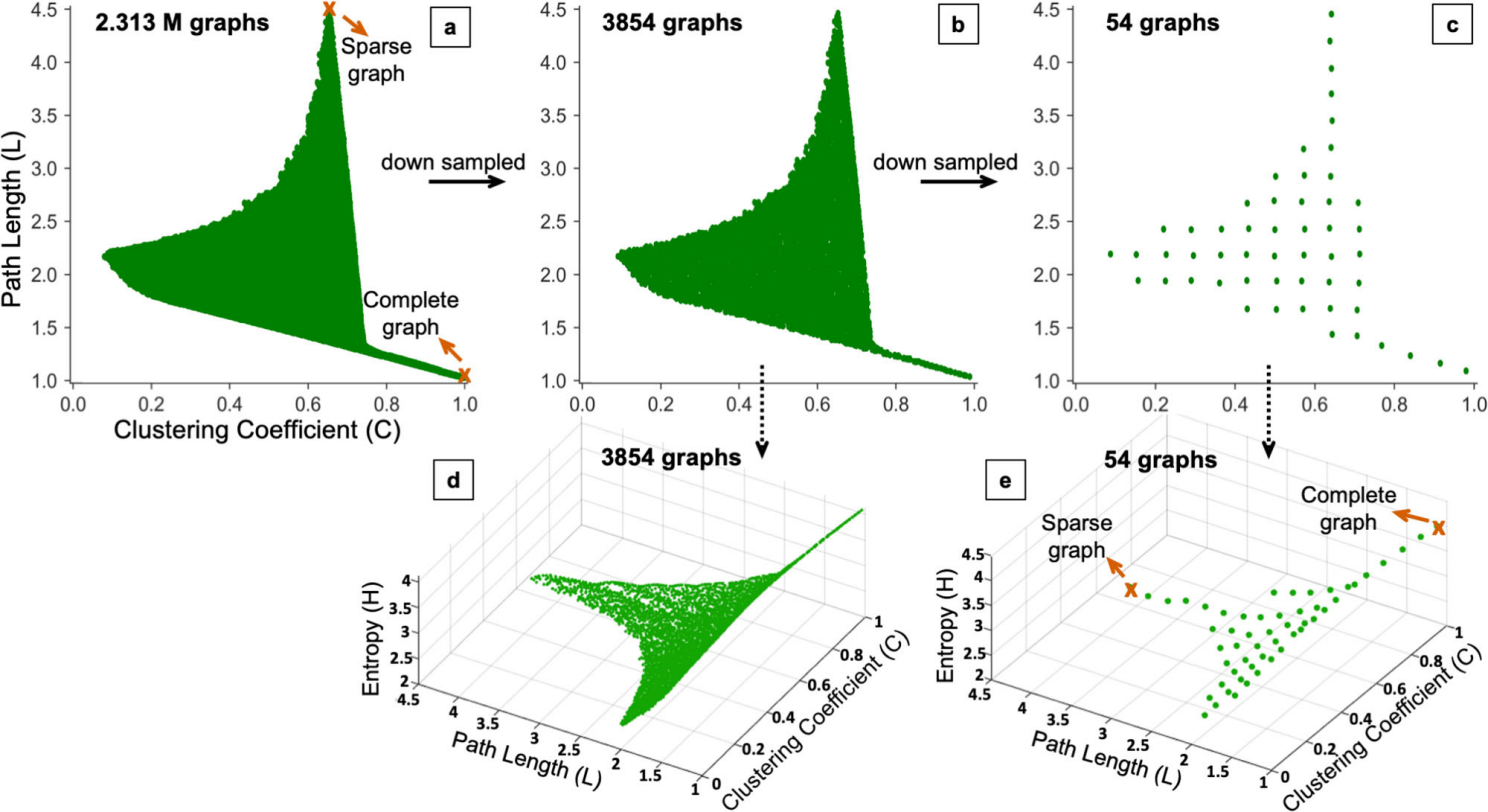
The graph design space for generating random graphs. **a**–**c** Show 2.313 M candidate graphs from Watts–Strogatz flex generator downsampled to 3854 and 54 graphs. **d**, **e** Show 3854 and 54 graphs in a 3-D space spanned by clustering coefficient (*C*), path length (*L*), and entropy (*H*). Samples of the complete and sparse graphs are identified in (**a**) and (**e**).

Being a sub-field of autoML, NAS is the process for searching suitable architectures of neural networks for a given task^35^. Design space is a component of NAS and is composed of a set of architectures of neural networks^36^. We use two graph measures, average path length (*L*) and clustering coefficient (*C*), for exploring the graph design space. Extensively used in prior works^37–39^, these measures smoothly span the whole design space of the random graphs as illustrated in Fig. 2. We generate 2.313 Million (M) candidate random graphs using Watts–Strogatz flex (WS-flex) graph generator for a range of *C* and *L* values as illustrated in Fig. 2a. We chose WS-flex because its graphs are superset of graphs generated by three classical methods including, WS, Erdős Rényi (ER), and Barabási-Albert (BA)^37,40,41^. We downsample 2.313 M candidate WS-flex graphs into coarser bins of 3854 and 54 graphs (Fig. 2b, c), where each bin has at least one representative graph. We visualize our candidate graphs using their average path length (*L*), clustering coefficient (*C*), and entropy (*H*). Entropy is a graph-theoretic measure for robustness and we visualize our design space spanned by (*C*, *L*, *H*), as depicted in Fig. 2d, e. Figure 2a, e also depicts the extreme cases of complete and sparse graphs. For a complete graph, we have (*C*, *L*, *H*) = (1.0, 1.0, 4.1).

We transform the downsampled 54 graphs into DANNs using the technique of relational graphs proposed by You et al.^9^. The same 54 graphs are transformed into multiple types of DANNs including, multilayer perceptrons (MLPs), convolutional neural networks (CNNs), and residual neural networks (ResNets). Because of the flexibility and generalizability of the relational graphs, our graphs-to-DANNs transformation framework can be translated into diverse architectures, including MLPs, CNNs, ResNets, EfficientNets, etc. We use four image classification datasets of varying complexity to train and evaluate DANNs built using 54 different graph structures. These datasets include Canadian Institute For Advanced Research for ten classes (CIFAR-10), hundred classes (CIFAR-100), Tiny ImageNet, and ImageNet^42–44^. The robustness of trained DANNs is quantified by subjecting these models to various levels and types of natural and malicious noise. We used three types of additive noise, Gaussian, Speckle, and Salt&Pepper. For malicious noise, we employ three different adversarial attacks with varying severity levels. These include Fast Gradient Sign Method (FGSM)^45^, Projected Gradient Descent (PGD)^46^, and Carlini Wagner (CW)^47^.

### Performance trends of DANNs

**Fig. 3:**
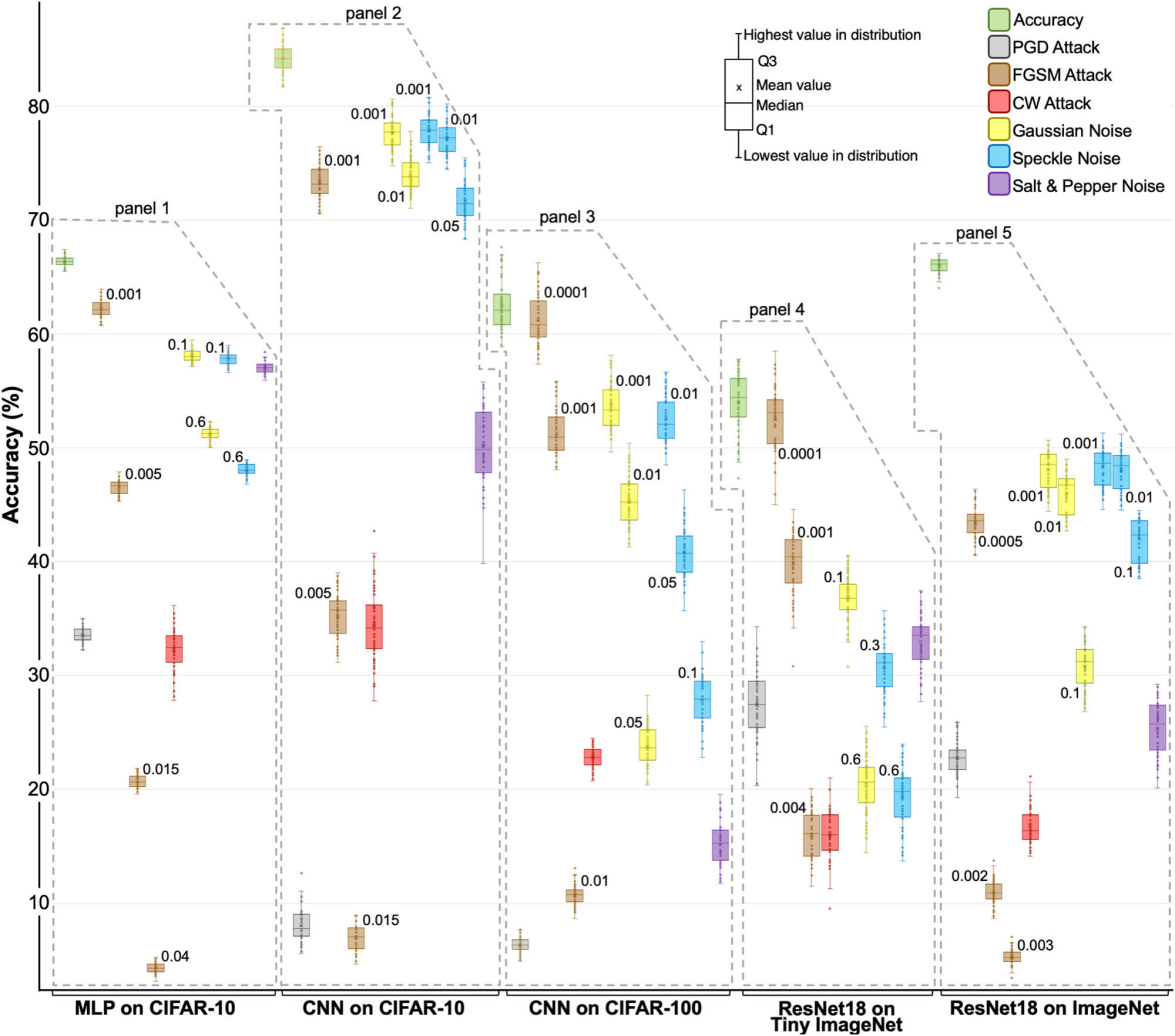
Test accuracies of different Deep Artificial Neural Networks (DANNs). The DANN models were trained and evaluated using image classification datasets under noisy conditions. Each box represents 54 different DANNs trained on the indicated dataset, i.e., 5-layer multilayer perceptrons (MLPs) trained on CIFAR-10, 8-layer convolutional neural networks (CNNs) on CIFAR-10, 8-layer CNNs on CIFAR-100, residual neural networks (ResNet-18) on Tiny ImageNet and ImageNet. Distribution bars for box plots are defined in the legend. DANNs are evaluated on clean images, adversarial examples (FGSM, PGD, CW attack), and noisy images (Gaussian, Speckle, Salt& Pepper noise). The severity levels for the FGSM, Gaussian, and Speckle noise are indicated on respective boxes. We observe a consistent decline in the predictive performance of all DANNs as the severity levels of adversarial attacks or natural noise increase.

Figure 3 presents predictive performance of different MLPs, CNNs, and ResNets built using 54 selected graphs and trained on four different image classification datasets. Performance evaluation of the trained DANNs is done using randomly selected 30 different sets of clean, adversarial, and noisy images. The test accuracy numbers presented in Fig. 3 are average values across all tests.

MLPs on CIFAR-10: Panel 1 of Fig. 3 presents test accuracies of 54 MLPs under different conditions. The average clean test accuracy is 66.3 ± 0.46%, which drops to 33.6 ± 0.64% under PGD attack and to 32.3 ± 1.9% for the CW attack. With FGSM attack levels of *ϵ* = [0.001, 0.005, 0.015, and 0.04], the test accuracy drops to [62.2 ± 0.72, 46.6 ± 0.6, 20.7 ± 0.54, 4.34 ± 0.44%]. For low noise level of natural or additive noise (${\sigma }_{\,{{\mbox{noise}}}}^{\,2}$ = 0.1), test accuracy under Gaussian noise is 58.1 ± 0.50% and under speckle noise 57.8 ± 0.52%. For high noise level (${\sigma }_{{{{{{{\mathrm{noise}}}}}}}}^{2}$ = 0.6), the test accuracy under Gaussian noise is 51.2 ± 0.55%, and under speckle noise 48.1 ± 0.55%. Under Salt&Pepper noise (salt vs. pepper = 0.5), the test accuracy is 57.06 ± 0.5%.

CNNs on CIFAR-10: Panel 2 of Fig. 3 shows the average test accuracies of 8-layer CNNs built from the same 54 candidate graphs. We observe that the average clean test accuracy for CNNs is 84.19 ± 1.26%, dropping to 8.07 ± 1.43% under PGD attack, and to 34.35 ± 3.12% under CW attack. We noticed similar trends for various levels of FGSM attacks, as well as for the Gaussian, speckle, salt&pepper noise.

CNNs on CIFAR-100: In panel 3 of Fig. 3, we present CNNs trained on CIFAR-100 dataset. The average test accuracy is 62.49 ± 2.24% for clean test dataset, which reduces to 6.35 ± 0.64% for the PGD attack, and 22.83 ± 0.94% for the CW attack. With FGSM attack levels of *ϵ* = [0.0001, 0.001, 0.01], the test accuracy is [61.19 ± 2.30%, 51.32 ± 2.06%, and 10.71 ± 0.83%]. For Gaussian noise levels of ${\sigma }_{\,{{\mbox{noise}}}\,}^{2}$ = [0.001, 0.01, 0.05], the test accuracy of CNNs is [53.75 ± 2.01%, 45.29 ± 2.12%, 23.76 ± 1.66%]. For speckle noise levels of ${\sigma }_{\,{{\mbox{noise}}}\,}^{2}$ = [0.01, 0.05, 0.1], the test accuracy is [52.54 ± 2.00%, 40.82 ± 2.11%, 27.79 ± 2.10%]. For salt&pepper noise, the test accuracy is 15.14 ± 1.81%. The drop in test accuracy for all cases is significantly more than that of CIFAR-10 dataset.

ResNet-18 on Tiny ImageNet: The panel 4 of Fig. 3 shows 54 different ResNets trained on Tiny ImageNet. The average clean test accuracy is 54.08 ± 2.54%, 27.45 ± 2.90% under PGD attack, and 16.00 ± 2.10% under CW attack. For the FGSM attack levels of *ϵ* = [0.0001, 0.001, and 0.004], the accuracy is [52.53 ± 2.85%, 39.86 ± 2.78%, 15.97 ± 2.04%]. For Gaussian noise levels of ${\sigma }_{\,{{\mbox{noise}}}\,}^{2}$ = [0.1, 0.6], the test accuracy is [36.81 ± 1.96%, 20.44 ± 2.47%]. For speckle noise levels of ${\sigma }_{\,{{\mbox{noise}}}\,}^{2}$ = [0.3, 0.6], the test accuracy is [30.73 ± 2.31%, 19.34 ± 2.53%]. For salt&pepper noise, the test accuracy is 33.00 ± 2.24%.

ResNet-18 on ImageNet: Panel 5 of Fig. 3 presents ResNets trained using ImageNet. Due to the large number of images available for training, the average clean test accuracy of all 54 ResNets-18 was 66.0 ± 0.62%, a significant improvement over Tiny ImageNet experiments (54.08 ± 2.54%). Under PGD attack, the test accuracy drops to 22.75 ± 1.39%, and to 16.78 ± 1.54% under CW attack. For the FGSM attack levels of *ϵ* = [0.0005, 0.002, 0.003], the accuracies are [43.47 ± 1.40%, 10.96 ± 1.09%, 5.27 ± 0.65%]. Similar trends are observed for the additive Gaussian and speckle noise under the ${\sigma }_{\,{{\mbox{noise}}}\,}^{2}$ = [0.001, 0.01, 0.1]. For salt&pepper noise, the test accuracy drops to 25.39 ± 2.35%.

Comparison of MLPs vs. CNNs on CIFAR-10: We observed that CNNs achieve higher accuracy on the clean test data as compared to MLPs on CIFAR-10 dataset. However, under adversarial conditions (FGSM, PGD, and CW attacks), the drop in the performance of CNNs is significantly higher than MLPs as shown in panels 1 and 2 of Fig. 3. The test accuracy drop is ~76% for CNNs compared to ~33% for MLPs under PGD attack. For the CW attack, the accuracy drop for CNNs is ~50% compared to ~34% for MLPs. The same trend was observed for all severity levels of the FGSM attack. Generally, as expected CNNs outperform MLPs under clean test conditions; however, MLPs are more robust to adversarial perturbations as compared to CNNs. We argue that the observed fragility of CNNs is linked to their weight sharing and shift-invariant characteristics, which was previously noted by Zhang et al.^48^.

### Robustness analysis

**Fig. 4:**
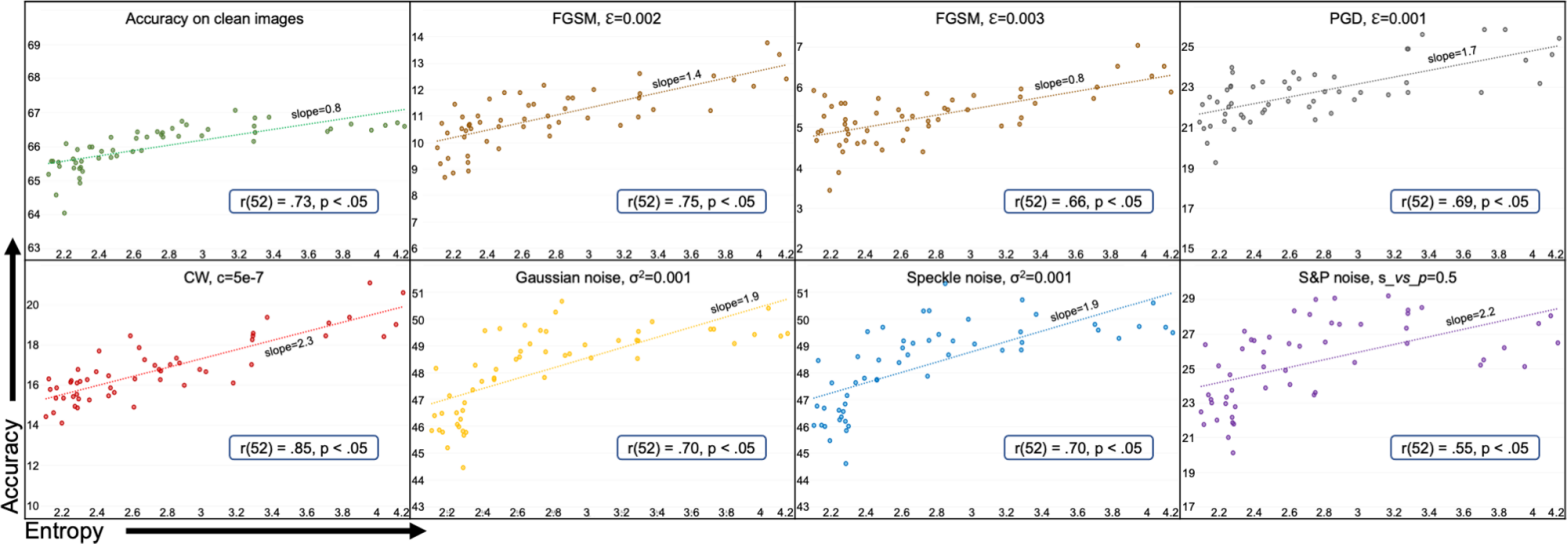
Test accuracy vs. entropy for ResNet-18 on ImageNet. Test accuracy is shown on the vertical axis and entropy (*H*) of the underlying graph is shown on the horizontal axis. The dots represent an average value calculated over five runs. The type and severity level of noise is shown on the top of each subplot. Sub-plots also show trendlines and Pearson correlation coefficients (*r*) with *p*-value. We note significant positive correlation between graph entropy and the performance of the Deep Artificial Neural Networks for all cases.

**Fig. 5:**
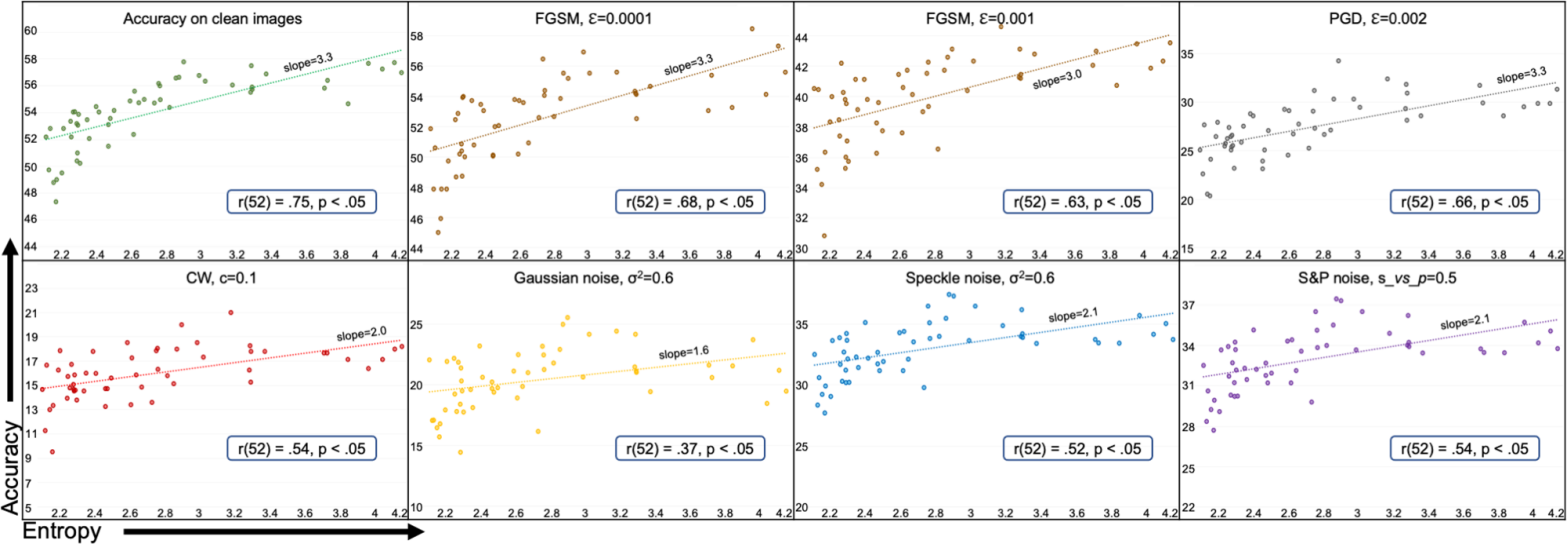
Test accuracy vs. entropy for ResNet-18 on Tiny ImageNet. Test accuracy is shown on the vertical axis and entropy (*H*) of the underlying graph is shown on the horizontal axis. The dots represent an average value calculated over five runs. We note a strong positive correlation between entropy and the accuracy of Deep Artificial Neural Networks for all types of noise cases.

**Fig. 6:**
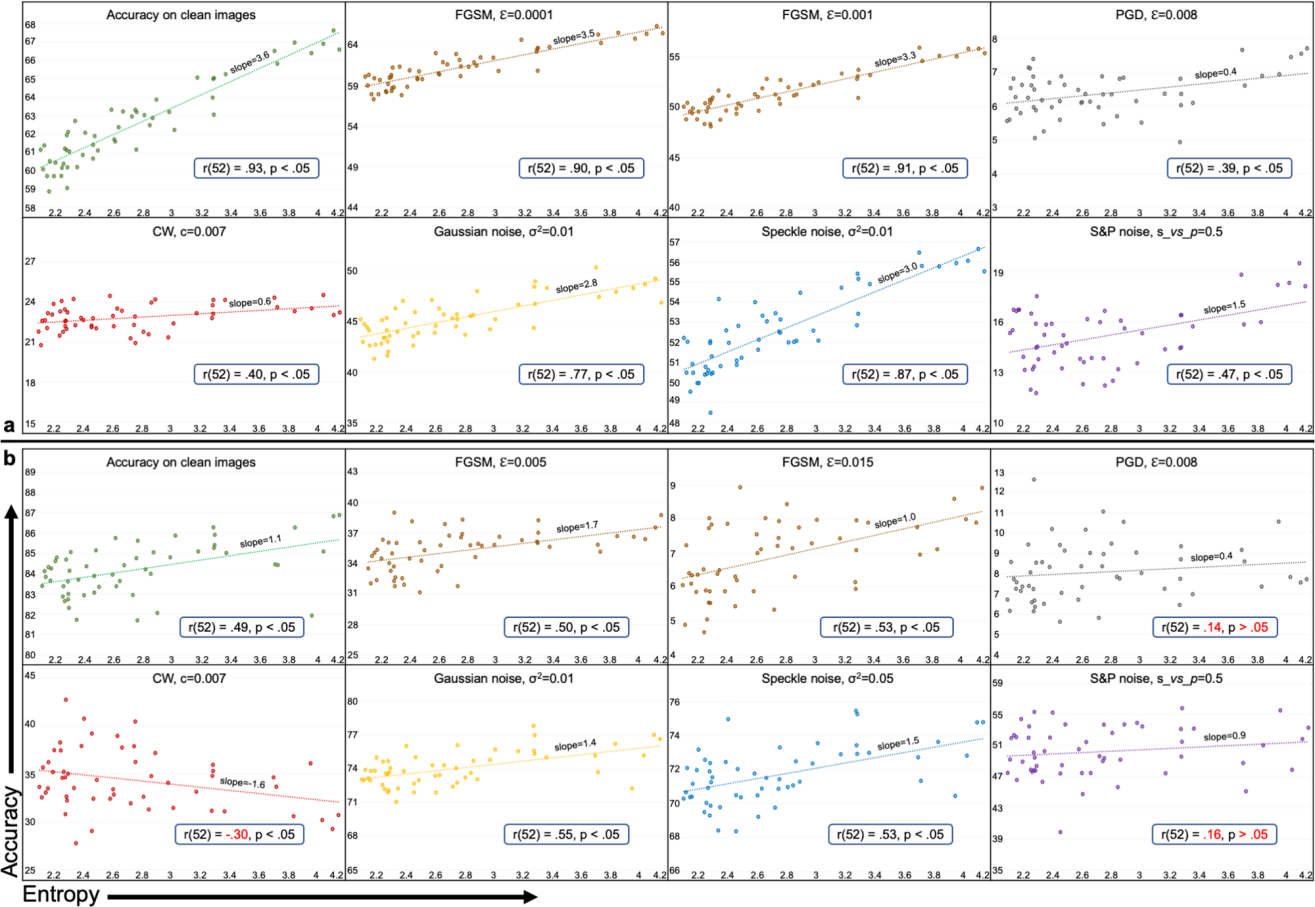
Accuracy vs. entropy for Convolutional Neural Networks (CNNs). CNNs were trained on **a** CIFAR-100 and **b** CIFAR-10 datasets and tested under various noisy conditions. The dots represent average test accuracy over 30 runs. The Pearson correlation *r* and corresponding *p* values between entropy and accuracy are also presented for each noise condition. The red text color shows correlation values that are not significant. For the same 8-layer CNNs, the entropy-robustness correlation values increase with the task complexity, that is, relatively higher correlation values are observed for CIFAR-100 as compared to CIFAR-10 dataset for all noise conditions.

Our work is a cross-pollination between graph theory and deep learning. We attempt to link the robustness of graphs underlying the architectures of DANNs to their performance against noise and adversarial attacks. On the graph theory side, we use entropy and Ollivier-Ricci curvature to quantify the robustness of graphs. These graphs, in turn, are used to build architectures of DANNs. On the deep learning side, we train these DANNs and quantify their robustness using test accuracy against various types of noise and adversarial attacks. Entropy and Ollivier-Ricci curvature have been extensively studied in the NetSci. These measures have been shown to capture the robustness of cancer networks^24,25^, track changes in brain networks caused by age and ASD^27^, explain cognitive impairment in patients with Multiple Sclerosis^28^, identify financial market’s fragility^26^, and detect communities in complex social networks^33^. We study the robustness of DANNs and establish the statistical correlation of the observed robustness with entropy and curvature. The correlation results for entropy of graphs and robustness of DANNs for different datasets are given in Figs. 4, 5, and 6. The correlation results between the robustness of DANNs and graph measures such as curvature, average degree, and global efficiency are provided in Supplementary Notes 3, 4, and 5, respectively.

ResNet-18 on ImageNet and Tiny ImageNet: Fig. 4 presents 54 ResNet-18 DANNs trained on ImageNet and tested on clean images, adversarial examples generated with FGSM, PGD, and CW attacks, and images with additive Gaussian, speckle, and salt&pepper noise. Each sub-plot shows entropy (*H*) of the underlying graph structure and the test accuracy of corresponding ResNet-18 under various conditions. The Pearson product-moment correlation coefficient values between entropy and accuracy along with *p* values are shown on each sub-plot. There was a very strong positive correlation between the two variables, *r* = 0.73, *n* = 54, *p* < 0.05 for the clean test dataset. We note similar behavior under PGD and CW attacks, that is, a strong correlation between entropy and accuracy exists, *r* = 0.69 for PGD and *r* = 0.85 for CW, *p* < 0.05 for both. Similarly, strong positive correlation trends exist for various severity levels of FGSM attack, Gaussian, speckle, and salt&pepper noise. The correlations in these experiments are all statistically significant (*p* < 0.05). In general, across all types of adversarial attacks and noises, the DANNs corresponding to graphs with higher entropy showed stronger robustness and vice versa. Additional results are provided in Supplementary Figs. S3 and S4.

Figure 5 presents test accuracy vs. entropy plots for 54 ResNet-18 models trained using Tiny ImageNet and tested under various noisy conditions. We observe a strong positive correlation between entropy and predictive performance under all noise conditions, except Gaussian noise where the correlation is moderate. However, there is a notable decrease in the Pearson product-moment correlation coefficient values in all noise categories compared to the same DANNs when trained and tested on ImageNet. As Tiny ImageNet is a subset of ImageNet with only 200 distinct classes instead of 1000, the observed decrease in the correlation may be linked to the reduction in complexity of the task, i.e., 200 classes instead of 1000.

CNNs on CIFAR-100 and CIFAR-10: In Fig. 6a, b, we present accuracy vs. entropy plots for the 54 8-layer CNNs trained on CIFAR-100 and CIFAR-10 datasets and tested under various noisy conditions. For the CIFAR-100 experiments, we observe very strong correlation between entropy and predictive performance except for CW (*r* = 0.40, *p* < 0.05), PGD (*r* = 0.39, *p* < 0.05) adversarial attacks, and salt&pepper noise (*r* = 0.47, *p* < 0.05). For CIFAR-10 dataset, there is a strong correlation between entropy and predictive performance except for the PGD, CW attacks and salt&pepper noise, which were not statistically significant.

We opine that the weak correlation between graph entropy and DANNs’ performance under PGD and CW attacks is due to the strong nature of PGD and CW attacks on relatively simple classification tasks of CIFAR compared to Tiny ImageNet and ImageNet. This opinion was strengthened from the evaluation results of the CNNs on a more straightforward classification task of CIFAR-10. We observe that the correlation of entropy with the predictive performance of CNNs reduces for all categories. Moreover, the entropy’s correlation with accuracy under CW attack becomes negative. Under PGD attack and salt&pepper noise, it becomes insignificant with *p* > 0.05 as highlighted by the red text in respective subplots of Fig. 6. Additional results are provided in Supplementary Figs. S5 and S6.

### Effect of task and model complexity

**Fig. 7:**
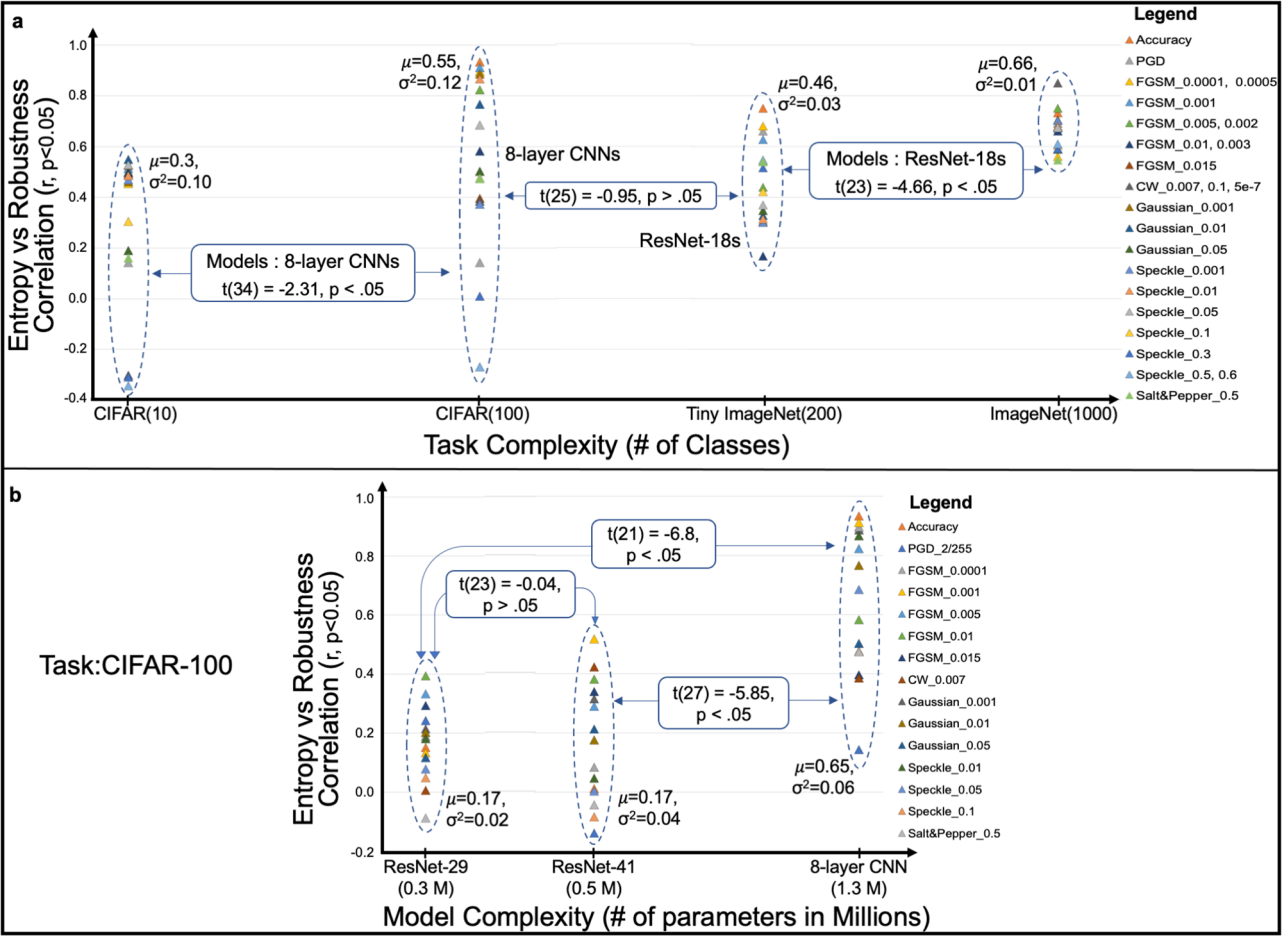
Effect of task and model complexity on entropy-robustness relationship. **a** Entropy-robustness correlation coefficient (vertical axis) is plotted against the number of classes (horizontal axis) in datasets. As the task becomes complex, entropy becomes significantly more correlated to the robustness of Deep Artificial Neural Networks (DANNs). **b** The entropy-robustness correlation coefficient is plotted against the number of model parameters for different DANNs (ResNet-29, ResNet-41, and CNNs) using CIFAR-100 dataset. For the same task, as the number of model parameters increases, the entropy-robustness correlation increases significantly (*p* < 0.05).

We observed that DANNs’ robustness, evaluated under noisy conditions, and the robustness of underlying graph structures, quantified using entropy, are strongly correlated. Moreover, this correlation has a strong dependence on the complexity of the model and/or the dataset. In our settings, the model complexity refers to the number of parameters in the model, and the task complexity refers to the number of classes in the dataset. As the complexity of the task and/or model increases, the correlation between robust performance and entropy of DANNs increases, as shown in Fig. 7.

In Fig. 7a, models evaluated for the 10-class and 100-class tasks are 8-layer CNNs. For 200-class and 1000-class tasks, ResNet-18 models were evaluated. We note that for the same 8-layer CNNs, increasing the complexity of the task (from 10 classes of CIFAR-10 to 100 classes of CIFAR-100) results in increase in the correlation values as noted by the Student’s t-test (*t* = −2.31, *n* = 34, *p* < 0.05). The same holds true for increasing the task complexity from 200 classes of Tiny ImageNet to 1000 classes of ImageNet and using the same ResNet-18 models (*t* = −4.66, *n* = 23, *p* < 0.05). Comparing two different models evaluated on separate tasks, we notice insignificant increase in the correlation values (i.e., p > 0.05) as in the case of 8-layer CNN models evaluated on CIFAR-100 dataset versus ResNet-18 models evaluated on Tiny ImageNet dataset. While Student’s t-test is used to compare two related samples, we used the F-test to see the equality of the two unrelated populations (CNNs vs. ResNet-18s) for performance variance on separate tasks. We observe that there is significance difference between variances of the two analyzed sets of experiments, *F*(14, 14) = 2.61, *p* < 0.05. In Fig. 7b, we present the effect of increasing the model complexity measured by the number of parameters against the entropy-robustness correlation. We observe that for the same CIFAR-100 dataset, as the model complexity increases from ~0.3 M parameters in ResNet-29 to ~1.3 M in CNN, the entropy-robustness correlation increases significantly (*t* = −6.8, *n* = 23, *p* < 0.05). Similarly, the entropy-robustness correlation increases significantly (*t* = −5.85, *n* = 27, *p* < 0.05) when model complexity increases from ~0.5 M parameters in ResNet-41 to ~1.3 M in CNN for CIFAR-100 task. We also note that this increase in significance is large when difference between the number of parameters between models is large. Our analysis of DANNs’ robustness show that a correlation exists between graph entropy and robustness of DANNs, and this correlation has a strong dependence on the complexity of task and model.

We summarize our results and robustness analysis here. The graph structural entropy of complex DANNs, like ResNets, is a strong indicator of their robustness against all of the analyzed additive noise and adversarial attacks on complex tasks such as 1000-class ImageNet. As we reduced the task complexity to 200-class Tiny ImageNet, the entropy-robustness relationship decreased for all categories of noise and adversarial attacks. Similarly, graph entropy of relatively simple DANNs, like 8-layer CNNs, is a strong indicator of robustness against relatively simple adversarial attacks, like FGSM, and all types of additive noise on the CIFAR-100 task. But under strong adversarial attacks, such as PGD and CW, the entropy-robustness relationship reduces for 8-layer CNNs. Similar trends were observed when testing the same 8-layer CNNs on the CIFAR-10 task. The only limitation we encountered in our analysis was that of MLPs trained and evaluated over CIFAR-10 dataset, as given in the Supplementary Note 2 and Supplementary Fig. S2.

## Discussion

In this work, we have shown that graph structural properties such as entropy and curvature can quantify the robustness of DANNs before training. We calculated entropy and curvature of a set of random graphs, which were later transformed into architectures of different types of DANNs. The DANNs were trained and their robustness was evaluated using different types of natural and adversarial noise. We noted that the robustness of trained DANNs was highly correlated with their graph measures of entropy and curvature. We also noted that the said correlations were even stronger for relatively large models and complex tasks.

Currently various autoML and NAS techniques are being developed to search for accurate model architectures for the given datasets and/or tasks^10–12,49,50^. We argue that for many mission-critical applications, the robustness of these models is equally or in some cases more important than accuracy. However, there are currently no assured ways of estimating the robustness of DANNs in the graph design space except training and testing the candidate DANNs in the deep learning domain. We suggest that the users of autoML/NAS techniques should incorporate entropy and Ollivier-Ricci curvature information into their search framework. Using the graph representation of DANNs within the defined search space, the structure and associated topological properties of DANNs, such as entropy and curvature can be studied. Integrating these structural measures with the existing performance criterion shall enable the autoML/NAS algorithms to quantitatively and qualitatively select robust DANNs instead of exhaustively searching through all possible candidates. The users and autoML/NAS algorithms can directly identify and choose the most robust model out of all the models that meet the accuracy criteria set by the user. Such a practice would allow users or autoML/NAS algorithms to choose accurate as well as robust DANNs keeping in view the application area of the machine learning model. In Supplementary Note 9, we have given an algorithm as a guideline for selecting the robust architecture of a DANN for a given design space.

We have focused our analyses in this work on the computer vision application. However, there are a plethora of other applications and tasks where robustness quantification of DANNs is important^51–54^. A possible future direction is to extend the presented analysis to more complex applications (e.g., natural language processing, graph data, and Deep Chip industry^55–59^) and larger models (e.g., Transformers, and Vision Transformers^60,61^). Given our current analysis, we anticipate that for the larger datasets, complex tasks, and huge models, the graph robustness measures will be even more relevant and will help users/autoML/NAS algorithms find robust DANN architectures.

## Methods

We start by presenting the techniques we employed for generating random graphs in the graph theory domain. Next, we describe the graph-theoretical properties used in our experiments to study random graphs. These graph measures are needed to study the structural information of the random graphs. Next, we provide details on transformations for building DANN architectures from random graphs and training these DANNs for various computer vision classification tasks. Finally, we present the multiple conditions, including natural noise and adversarial attacks that we used to evaluate the trained DANNs and quantify their robustness.

### Generating random graphs

Random graphs are extensively used in percolation studies, social sciences, brain studies, and deep learning to understand the behavior of natural systems and DANNs^8,39,40,62,63^. We used random graphs, called relational graphs, employed recently in deep learning^9^.

Relational graphs: A recent study used relational graphs and showed that the performance of a DANN can be quantified using its graph properties such as clustering coefficient and path length^9^. The relational graphs are generated through the WS-flex graph generator. WS-flex is a generalized version of the WS model having same-degree constraint relaxed for all nodes. Parameterized by *N* nodes, *K* average degree, and *P* rewiring probability, we represent these graphs by WS-flex(*N*, *K*, *P*). For the graph generator, we use notation *g*(*θ*, *s*), where *g* is the generator (for example, WS-flex), *θ* represents parameters (*N*, *K*, *P*), and *s* is the random seed. It is important to note that WS-flex(*N*, *K*, *P*) graph generator encompasses the design space of all the graphs generated by the three classical families of random graph generators, including WS, ER, and BA^9,37,40,41^.

### Graph-theoretic measures

Average path length (*L*). It is a global graph measure defined as the average shortest path distance between any pair of graph nodes. It depicts the efficiency of the graph with which information is transferred through the nodes^64^. Small values of *L* indicate that the graph is globally efficient, and the information is effectively exchanged across the whole network and vice versa. Let *G* be an unweighted directed graph having *V*, a set of *n* vertices {*v*_1_, *v*_2_, . . . , *v*_*n*_} ∈ *V*. Let *d*(*v*_1_, *v*_2_) be the shortest distance between *v*_1_, *v*_2_ and *d*(*v*_1_, *v*_2_) = 0 if *v*_2_ is unreachable from *v*_1_. Then, average path length *L* is defined as,

1 $$L=\frac{1}{n(n-1)}\mathop{\sum}\limits_{i\ne j}d({v}_{i},{v}_{j}).$$

Clustering coefficient (*C*). Clustering coefficient is a measure of the local connectivity of a graph. For a given node *i* in a graph, the probability that all its neighbors are also neighbors to each other is called clustering coefficient. The more densely interconnected is the neighborhood of a node, the higher is its measure of *C*. Large value of *C* is linked with the resilience of the network against random network damage^65^. The small-worldness of networks is also assessed by *C*^66^. For a node *i* with degree *k*_*i*_, clustering coefficient *C*_*i*_ is defined as,

2 $${C}_{i}=\frac{2{d}_{i}}{{k}_{i}({k}_{i}-1)},\qquad \quad 0\le {C}_{i}\le 1.$$

where *d*_*i*_ is the number of edges between the *k*_*i*_ neighbors of node *i*.

Graph spectral measures. The spectral measures focus on eigenvalues and eigenvectors of the associated graph adjacency and Laplacian matrices. We use topological entropy and Ollivier-Ricci curvature.

- Topological entropy (*H*). Entropy of graph *G* having adjacency matrix *A*_*G*_, is the logarithm of the spectral radius of *A*_*G*_, i.e., logarithm of the maximum of absolute values of the eigenvalues of *A*_*G*_^67^.
  3 $$H=\log ({\lambda }_{{A}_{G}}).$$
- Ollivier-Ricci curvature (ORC). It is the discrete analog of the Ricci curvature^68,69^. From the many alternatives of Ricci curvature^70^, we use the definition presented by Farooq et al. ^27^ (see Fig. 6 of ref). Let (*X*, *d*) be a geodesic (a curve representing the shortest path between two points on a surface or in a Riemannian manifold) metric space having a family of probability measures {*p*_*x*_: *x* ∈ *X*}. Then, ORC *κ*_*ORC*_(*x*, *y*) along the geodesic connecting *x* and *y* is,
  4 $${\kappa }_{ORC}(x,y)=1-\frac{{W}_{1}({p}_{x},{p}_{y})}{d(x,y)},$$
  where *W*_1_ is the earth mover’s distance (Wasserstein-1 metric), and *d* is the geodesic distance on the space. Curvature is directly proportional to the robustness of the network. The larger the curvature, the faster will be the return to the original state after perturbation. Smaller curvature means slow return, which is also called fragility^27^.

Robustness and Fragility. We now provide the notion of robustness and fragility used in this paper. Fluctuation theorem^71^ gives the concept of measuring a network’s potential of returning to its “relaxed” and unperturbed state when subjected to some random perturbation. Let *p*_*γ*,*α*_(*t*) denote the probability that under some perturbation *γ* at time *t*, the observable mean deviation of the network from its relaxed state is greater than *α*. The rate *R* at which a dynamic system returns to its original state after perturbation is given by the following function,

5 $$R:= \mathop{\lim }\limits_{t\to \infty }\left(-\frac{1}{t}\log {p}_{\gamma ,\alpha }(t)\right).$$

Here, a large value of *R* denotes a prompt return to relaxed state after a small perturbation(*γ*), called the network robustness, whereas, a small *R* means slow return from a large perturbation(*γ*), called the network fragility. In the field of thermodynamics, entropy is closely related to the rate function *R* from large perturbations^71,72^. Fluctuation theorem^71,73^ states that, given random perturbations to the network, change in system entropy Δ*H* is positively correlated to change in robustness Δ*R*, and negatively correlated to change in fragility Δ*F*, (since Δ*R* ≔ −Δ*F*).

6 $$\Delta H\times \Delta R \, > \, 0,$$

7 $$\Delta H\times \Delta F\,\le\, 0.$$

Entropy Δ*H* and curvature Δ*κ*_ORC_ are also positively correlated (see Equation (7) of Tannenbaum et al. ^24^), that is,

8 $$\Delta H\times \Delta {\kappa }_{{{{{\rm{ORC}}}}}}\, > \, 0.$$

From Eqs. (6) and (8), we see that graph curvature and robustness are also positively correlated,

9 $$\Delta {\kappa }_{{{{{\rm{ORC}}}}}}\times \Delta R \, > \, 0.$$

Equations (6) and (9) are the primary motivation in this work to study the curvature and entropy of deep neural networks.

### From graphs to DANNs

Let *G* = (*V*, *ε*) be a graph having node-set *V* = {*v*_1_, *v*_2_, . . . , *v*_*n*_}, where node *v* has feature vector **x**_*v*_, and edge set *ε* = {(*v*_*i*_, *v*_*j*_)∣*v*_*i*_, *v*_*j*_ ∈ *V*}. The neighborhood of node *v* is defined as *N*(*v*) = {*u*∣(*u*, *v*) ∈ *ε*}. To transform the graphs into DANNs, we adopt the concept of neural networks as relational graphs^9^. In relational graph, a single node represents one input channel and one output channel. Edge in the relational graph represents a message exchange between the two nodes it connects. The message exchange is a message function having node feature **x**_*v*_ as input and a message-aggregation function as output. The aggregation function takes a set of messages as input and gives an updated node feature as output. One iteration of this process is one round of message exchange. At each round, each node sends messages to its neighbors, receives messages from all the neighbors, and aggregates them. At each edge, message transformation occurs through a message function *f*(.), followed by summation at each node through an aggregation function *F*(.). The *i*th message exchange round between nodes *v* and *u* can be expressed as,

10 $${{{{{{{{\bf{x}}}}}}}}}_{v}^{(i+1)}={F}^{(i)}(\{{f}_{v}^{(i)}({{{{{{{{\bf{x}}}}}}}}}_{u}^{(i)}),\forall u\in N(v)\}).$$

You et al. have shown that Eq. (10) is the general definition of message exchange that can be used to instantiate any neural architecture^9^. We generate MLP, CNN, ResNet-18, and ResNet-29 for each of the 54 random graphs generated from the WS-flex generator. We have illustrated the graph-to-DANN transformation for a 64-node complete graph, generated from the WS-flex generator, to a 5-layer MLP, 8-layer CNN, and ResNet-18 models in the Supplementary Note 1 and Supplementary Fig. S1.

The same 54 WS-flex random graphs were transformed into a total of 216 DANNs having 54 neural networks in each of the four categories (MLP, CNN, ResNet-18, and ResNet-29). MLPs were trained on CIFAR-10 dataset, whereas, the CNNs were used for training on CIFAR-10 and CIFAR-100 datasets. The same ResNets-18 were used for training on ImageNet and Tiny ImageNet datasets. The baseline architectures have a complete graph structure for each architecture category. To ensure the consistency of our results, we trained each MLP and CNN five times and ResNets one time on respective datasets. The results reported in this paper are average values calculated for thirty different inferences over random test inputs for each MLP and CNN, whereas, five random test inference runs for each ResNet. List of frameworks and hyperparameters used in our experiments are provided in Supplementary Note 6. The compute resources and wall clock times are given in Supplementary Note 7.

### Datasets

We used four different image classification datasets for our experiments that allowed us to train DANNs of different sizes on tasks that varied in their complexity. We used 10-class CIFAR-10^42^ dataset to train MLPs and CNNs. CIFAR-100^42^ dataset having 100 classes was used to train CNNs and ResNet-29. Both datasets have 50,000 training images and 10,000 validation images. To further scale our experiments, we trained ResNet-18 on the Tiny ImageNet^43^ dataset having 200 classes. Each class in Tiny ImageNet has 500 training images and 50 validation images. We also trained ResNet-18 on the ImageNet^44^ dataset having 1000 classes, 1.2 M training images and 50,000 validation images.

### Robustness analysis

We assessed the robustness of DANNs against natural additive noise and malicious noise (adversarial attacks). First, we evaluated the models using clean test images from respective datasets. Then we fed DANNs with different test images corrupted with additive noise and adversarial attacks. It is important to note that we chose the severity levels of adversarial attacks and additive noise so that the predictive performance of DANNs is at the minimum greater than 3%. We observed at higher levels of noise, the performance would naturally drop to 0%, which was not helpful in our analysis. Moreover, different severity levels work on different datasets owing to the inherent features and attributes of the data.

Performance evaluation under adversarial attacks. We evaluated DANNs using adversarial examples generated from three different types of attacks, (1) FGSM^45^, (2) PGD^46^, and (3) CW^47^.

Consider a valid input *x*_0_ and a target class *y*_0_. It is possible to find *x* through non-random perturbation to *x*_0_ that changes a DANN’s prediction to some other *y*; such *x* is called an adversarial example. Given a loss function *J*(***x***;***w***), ***x***_0_ be the input to the model having parameter ***w***, the adversarial example ***x*** is created by the adversarial attack as,

11 $$\,{{\mbox{FGSM}}}\,:\quad \quad x={x}_{0}+\epsilon \cdot {{{{{{{\rm{sign}}}}}}}}({\nabla }_{x}J({x}_{0};w)),$$

12 $$\,{{\mbox{PGD}}}\,:\quad \quad {x}^{t+1}={\Pi }_{x+B}\{{x}^{t}+\alpha \cdot sign({\nabla }_{x}J({x}^{t};w))\},$$

13 $${{{{{{{\rm{CW}}}}}}}}:\quad \quad \mathop{\min }\limits_{x}\parallel x-{x}_{0}{\parallel }^{2}+c\cdot \max \{(\mathop{\max }\limits_{i\ne j}\{{g}_{j}(x)\}-{g}_{t}(x)),0\}.$$

In Eq. (11), *ϵ* is the severity level of the attack and should be small enough to make the perturbation undetectable. In Eq. (12), *x*^*t*^ is an adversarial example after *t*-steps, *α* is the step-size, Π_*x*+*B*_ refers to the projection operator for each input *x* having a set of allowed perturbations *B* chosen to capture the perceptual similarity between images. In Eq. (13), *c* > 0 is the attack magnitude, *i* is the input class, and *j* is the target class. FGSM and PGD have the *l*_*∞*_-distance metric, whereas CW, a regularization-based attack, has *l*_2_-distance metric in our analysis.

For the FGSM attacks, we used eighteen severity levels, *ϵ* = [0.0001, 0.0005, 0.001, 0.0015, 0.002, 0.0025, 0.003, 0.004, 0.005, 0.01, 0.015, 0.02, 0.025, 0.04, 0.045, 0.06, 0.08, and 0.3]. For the PGD attacks on CIFAR datasets, we used $\max (B)=0.008$, *α* = 2/255, and *t* = 7. For the Tiny ImageNet dataset, we used $\max (B)=0.002$, *α* = 2/255, and *t* = 10, and for the ImageNet dataset, we used $\max (B)=0.001$, *α* = 2/255, and *t* = 10. For the CW attacks on CIFAR datasets, we used *c* = 0.007 and steps = 100. For the Tiny ImageNet dataset, we used *c* = 0.01, steps = 100, whereas for the ImageNet dataset, we used *c* = 5e−7 and steps = 100.

Testing under additive noise. We used three different types of noise to generate corrupt images for all the datasets, (1) Gaussian, (2) speckle, and (3) salt&pepper noise. For each noise type, we used different levels of corruption quantified by the variance and monitored the performance drop. The noise variance used in our experiments for the Gaussian and speckle noise types are *σ*^2^ = [0.001, 0.01, 0.05, 0.1, 0.2, 0.3, 0.4, 0.5, and 0.6]. For the salt&pepper noise type, we used the maximum ratio of salt vs. pepper = 0.5, where salt changes a pixel value to 1 randomly, and pepper changes a pixel value to 0 randomly, in the input image. Sample images for each dataset used in our experiments, with noise types and levels are shown in Supplementary Note 8, Supplementary Figs. S8 and S9.

### Statistical analysis

We conducted various statistical tests to ascertain the significance of our analysis. We computed the Pearson product-moment correlation coefficient to assess the relationship between adversarial accuracy and the graph robust structural properties. We also computed the Pearson product-moment correlation coefficient between different structural graph-theoretic measures as shown in Supplementary Fig. S7. For reference, Pearson product-moment correlation coefficient *r* ranges from −1 to +1, where larger the absolute value of *r*, the higher is the degree of correlation and stronger is the relationship between variables, and vice versa. Specifically, the absolute values of *r* = 0 indicates no relationship, 0 < *r* ≤ 0.3 indicates weak relationship, 0.3 < *r* ≤ 0.4 indicates a moderate relationship, 0.4 < *r* ≤ 0.7 indicates strong relationship, *r* > 0.7 indicates very strong relationship, and *r* = 1 indicates perfect relationship. We used the Student’s t-test to establish that the average of the correlations between entropy and robustness for two types of datasets as well as two model types are statistically different. This analysis established how entropy is related to the increase in model size and task complexity. The significance level in all these analyses is set to 95%, i.e., *p* < 0.05 indicates statistically significant values.

### Supplementary information

Waqas_PR File

Supplementary Information

Description of Additional Supplementary Files

Supplementary Data

## Data Availability

The datasets used in this study are publicly available on following links: CIFAR-10 and CIFAR-100 (https://www.cs.toronto.edu/~kriz/cifar.html), Tiny ImageNet (https://www.kaggle.com/c/tiny-imagenet/overview), and ImageNet (https://www.image-net.org/). The source data of figures are given in Supplementary Data. Correspondence should be addressed to A.W.

## Code availability

For the simulations in deep learning domain, we have used PyTorch machine learning library, primarily developed by Facebook’s AI Research lab. The base-code for relational graph experiments (https://github.com/facebookresearch/graph2nn/) is under the MIT License with copyright(c)Facebook, Inc. and its affiliates. The calculations in graph domain and graph theoretic measures have been coded in Matlab software. Both of these code-packages (in PyTorch and Matlab) are published in the GitHub repository associated with this paper (https://github.com/Waasem/RobDanns).
