# Supplementary material for: Exploring robust architectures for deep artificial neural networks
Source: Commun Eng. 2022 Dec 17;1:46. doi: 10.1038/s44172-022-00043-2 (PMC10955826; doi:10.1038/s44172-022-00043-2)
Supplement: Supplementary file 2 — Supplementary Information [file 44172_2022_43_MOESM2_ESM.pdf]

Supplementary information - Exploring robust architectures for deep artificial neural networks

Asim Waqas, Hamza Farooq, Nidhal C. Bouaynaya, and Ghulam Rasool

*Corresponding author. E-mail: asim.waqas@moffitt.org;

Supplementary Note 1 Graph to DANN transformation

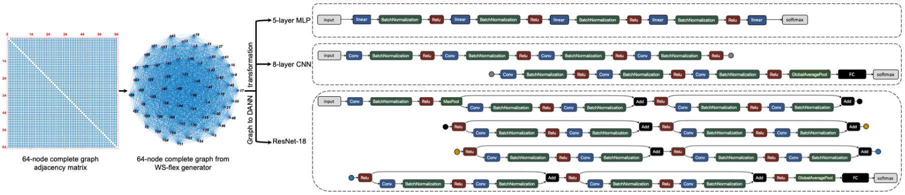

Supplementary Fig. S1 Schematic for graph to DANN transformation. 64-node complete graph generated from the WS-flex generator is shown along with its adjacency matrix. Using relational graph transformation, this complete graph is transformed into a 5-layer MLP, 8-layer CNN, and ResNet-18 model. The transformed DANNs are then trained and tested for the given task.

Supplementary Note 2 Further Results and Limitations of the Study

In our experiments with 5-layer MLPs trained and tested over CIFAR-10 dataset, we noticed that graph structural measures do not efficiently quantify the robustness of MLPs for low severity levels of adversarial attacks and additive noise. MLPs are very dense networks having no weight sharing. Each neuron in MLP has multiple edges across layers, making them fully connected (FC) networks. Under insults such as adversarial attack and natural noise, the MLPs are inherently robust because multiple neurons collectively contribute to the same task. MLPs depict superior accuracy for a given task than CNNs under a robust training regime [1]. We believe that because of the in-built robust nature of MLPs, the graph structural properties such as entropy and curvature do not significantly differentiate robust and fragile MLPs. Results of MLPs trained five times on the CIFAR-10 dataset and randomly evaluated thirty times are illustrated in Supplementary Fig. S2. The correlation between entropy and accuracy of MLPs is insignificant for most of the evaluation categories.

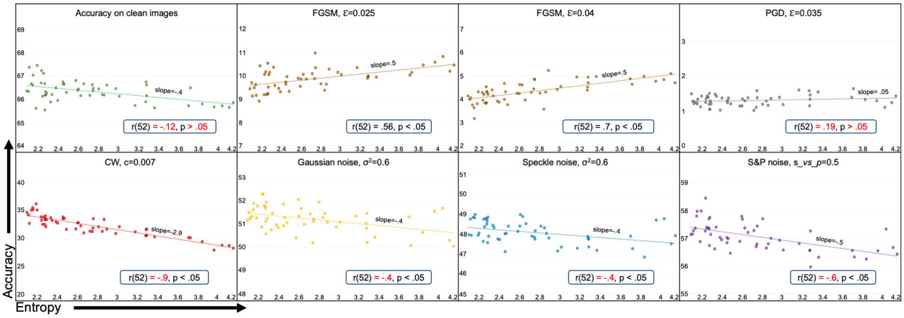

Supplementary Fig. S2 Test accuracy vs. entropy for MLPs trained on CIFAR-10 dataset. The correlations between entropy and test accuracy are insignificant for most of the evaluation categories.

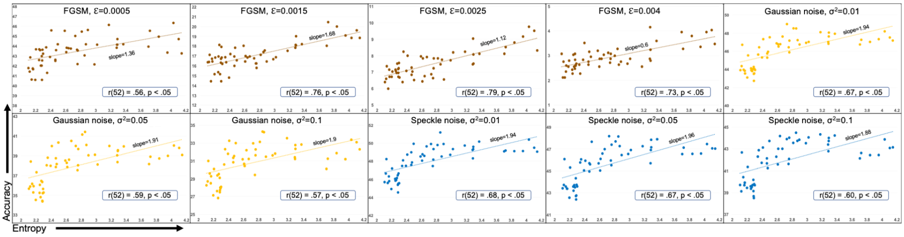

Supplementary Fig. S3 Additional results for ResNet-18 on ImageNet dataset. The experiments show strong positive correlation between entropy and test accuracy for all evaluation categories.

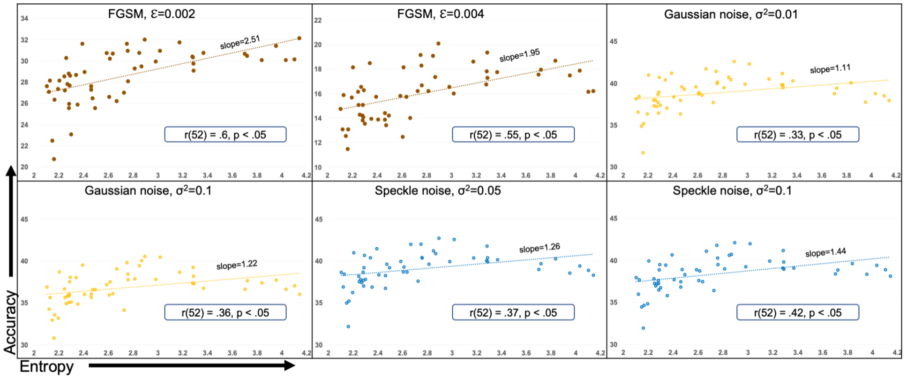

Supplementary Fig. S4 Additional results for ResNet-18 trained and evaluated on Tiny ImageNet dataset.

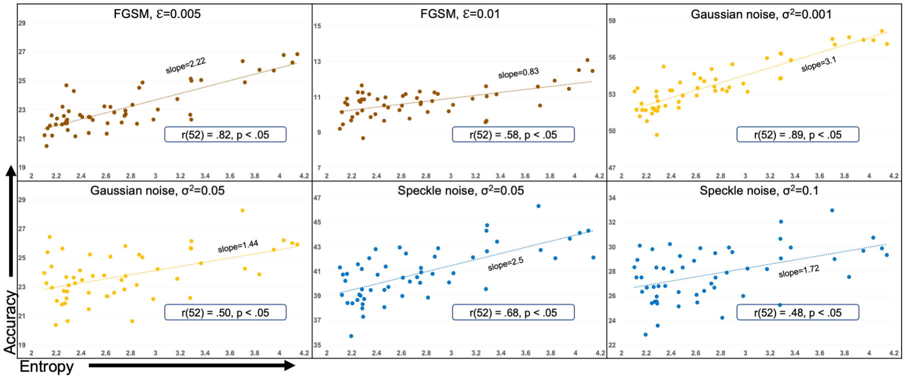

Supplementary Fig. S5 Additional results for robustness evaluation of CNN on CIFAR-100 dataset.

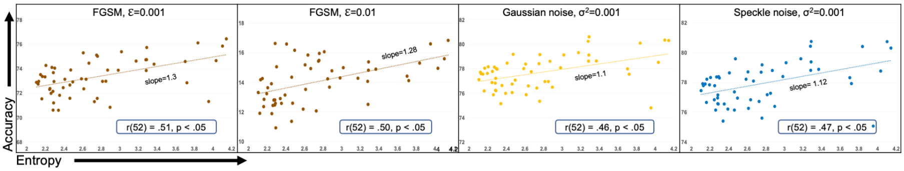

Supplementary Fig. S6 Additional results for robustness evaluation of CNN on CIFAR-10 dataset.

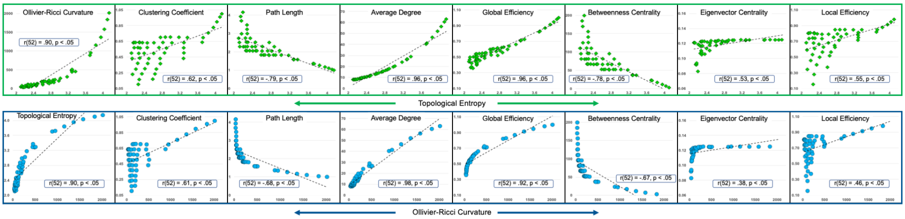

Supplementary Fig. S7 Correlation of Entropy and Curvature with other graph measures considered in our study. The Pearson correlation coefficient is shown in the inset box of each plot. Top row (in green color) shows the correlation of topological entropy with other considered graph measures. The bottom row (in blue color) depicts the correlation between curvature and considered graph properties. Consistent with the findings of previous studies in NetSci, the graph structural properties of entropy and curvature are better indicators of DANNs' robustness.

Supplementary Note 3 Curvature vs. test accuracy

Supplementary Table S1 Curvature vs. test accuracy

	ResNet-18			CNN	
**Metric**	ImageNet	Tiny ImageNet	**Metric**	CIFAR-100	CIFAR-10
Clean Accuracy	0.50	**0.55**	Clean Accuracy	**0.83**	0.45
FGSM(ϵ=.001)	**0.56**	0.49	FGSM(ϵ=.0001)	**0.79**	0.42
FGSM(ϵ=.002)	**0.68**	0.45	FGSM(ϵ=.001)	**0.83**	0.46
FGSM(ϵ=.003)	**0.64**	0.35	FGSM(ϵ=.005)	**0.81**	0.43
FGSM(ϵ=.004)	**0.69**	0.35	FGSM(ϵ=.01)	**0.64**	0.44
PGD(B=0.001)	**0.61**	-	FGSM(ϵ=.015)	0.45	**0.46**
PGD(B=.002)	-	0.48	PGD(B=.008)	0.52	0.07†
CW(c=5e-7)	**0.78**	-	CW(c=.007)	**0.36**	-0.33
CW(c=.1)	-	0.38			

	ResNet-18			CNN	
**Metric**	ImageNet	Tiny ImageNet	**Metric**	CIFAR-100	CIFAR-10
Gau(σ^2=.001)	**0.49**	0.08†	Gau(σ^2=.001)	**0.80**	0.41
Gau(σ^2=.01)	**0.45**	0.10†	Gau(σ^2=.01)	**0.66**	0.47
Gau(σ^2=.05)	**0.38**	0.13†	Gau(σ^2=.05)	**0.50**	0.12†
Gau(σ^2=.1)	**0.37**	0.14†	Spkl(σ^2=.01)	**0.76**	0.44
Spkl(σ^2=.001)	**0.49**	0.07†	Spkl(σ^2=.05)	**0.58**	0.47
Spkl(σ^2=.01)	**0.46**	0.09†	Spkl(σ^2=.1)	**0.43**	0.23†
Spkl(σ^2=.05)	**0.45**	0.14†	S&P(*ratio*=.5)	**0.59**	0.15†
Spkl(σ^2=.1)	**0.36**	0.21†			
S&P(*ratio*=.5)	0.33	**0.34**			

Pearson correlation coefficient between graph curvature and test accuracy of DANNs. All values except (†) are significant, r(52), p<0.05. The bold font indicates the better accuracy of the same DANN on one dataset compared to the other dataset. † denotes insignificant correlation values, r(52), p>0.05. These results indicate that curvature can quantify the robustness of DANNs, especially in complex tasks and bigger models.

Supplementary Note 4 Average degree vs. test accuracy

Supplementary Table S2 Average degree vs. test accuracy

	ResNet-18			CNN	
**Metric**	ImageNet	Tiny ImageNet	**Metric**	CIFAR-100	CIFAR-10
Clean Accuracy	0.60	**0.63**	Clean Accuracy	**0.89**	0.47
FGSM(ϵ=.001)	**0.61**	0.54	FGSM(ϵ=.0001)	**0.85**	0.44
FGSM(ϵ=.002)	**0.71**	0.51	FGSM(ϵ=.001)	**0.88**	0.49
FGSM(ϵ=.003)	**0.67**	0.44	FGSM(ϵ=.005)	**0.83**	0.46
FGSM(ϵ=.004)	**0.72**	0.44	FGSM(ϵ=.01)	**0.63**	0.47
PGD(B=0.001)	**0.65**	-	FGSM(ϵ=.015)	0.45	**0.49**
PGD(B=.002)	-	0.55	PGD(B=.008)	0.48	0.09†
CW(c=5e-7)	**0.82**	-	CW(c=.007)	**0.37**	-0.33
CW(c=.1)	-	0.45			

	ResNet-18			CNN	
**Metric**	ImageNet	Tiny ImageNet	**Metric**	CIFAR-100	CIFAR-10
Gau(σ^2=.001)	**0.58**	0.15†	Gau(σ^2=.001)	**0.85**	0.44
Gau(σ^2=.01)	**0.53**	0.17†	Gau(σ^2=.01)	**0.71**	0.51
Gau(σ^2=.05)	**0.46**	0.20†	Gau(σ^2=.05)	**0.51**	0.14†
Gau(σ^2=.1)	**0.45**	0.22†	Spkl(σ^2=.01)	**0.82**	0.46
Spkl(σ^2=.001)	**0.57**	0.15†	Spkl(σ^2=.05)	**0.63**	0.50
Spkl(σ^2=.01)	**0.54**	0.16†	Spkl(σ^2=.1)	**0.46**	0.26†
Spkl(σ^2=.05)	**0.54**	0.22†	S&P(*ratio*=.5)	**0.56**	0.16†
Spkl(σ^2=.1)	**0.45**	0.28			
S&P(*ratio*=.5)	**0.42**	**0.42**			

Pearson correlation coefficient between average degree of graphs and test accuracy of DANNs. All values except (†) are significant, r(52), p<0.05. Bold text indicates better accuracy of the same DANN evaluated on one dataset compared to the other dataset. † mark the insignificant correlation values, r(52), p>0.05. Average degree of graphs are also related to robustness of DANNs.

Supplementary Note 5 Global efficiency vs. test accuracy

Supplementary Table S3 Global efficiency vs. test accuracy

	ResNet-18		CNN		
**Metric**	ImageNet	Tiny ImageNet	**Metric**	CIFAR-100	CIFAR-10
Clean Accuracy	0.73	**0.78**	Clean Accuracy	**0.93**	0.45
FGSM(ϵ=.001)	**0.71**	0.68	FGSM(ϵ=.0001)	**0.90**	0.43
FGSM(ϵ=.002)	**0.80**	0.65	FGSM(ϵ=.001)	**0.90**	0.49
FGSM(ϵ=.003)	**0.67**	0.50	FGSM(ϵ=.005)	**0.83**	0.48
FGSM(ϵ=.004)	**0.75**	0.54	FGSM(ϵ=.01)	**0.58**	0.48
PGD(B=0.001)	**0.71**	-	FGSM(ϵ=.015)	0.34	**0.50**
PGD(B=.002)	-	0.66	PGD(B=.008)	0.45	0.15†
CW(c=5e-7)	**0.85**	-	CW(c=.007)	**0.43**	-0.27
CW(c=.1)	-	0.52			

	ResNet-18		CNN		
**Metric**	ImageNet	Tiny ImageNet	**Metric**	CIFAR-100	CIFAR-10
Gau(σ^2=.001)	**0.71**	0.36	Gau(σ^2=.001)	**0.89**	0.43
Gau(σ^2=.01)	**0.67**	0.38	Gau(σ^2=.01)	**0.78**	0.52
Gau(σ^2=.05)	**0.59**	0.39	Gau(σ^2=.05)	**0.51**	0.17†
Gau(σ^2=.1)	**0.55**	0.39	Spkl(σ^2=.01)	**0.88**	0.46
Spkl(σ^2=.001)	**0.70**	0.35	Spkl(σ^2=.05)	**0.70**	0.51
Spkl(σ^2=.01)	**0.68**	0.37	Spkl(σ^2=.1)	**0.51**	0.28
Spkl(σ^2=.05)	**0.67**	0.42	S&P($ratio$=.5)	**0.48**	0.13†
Spkl(σ^2=.1)	**0.59**	0.46			
S&P($ratio$=.5)	**0.52**	**0.52**			

Pearson correlation coefficient between global efficiency measure of graphs and test accuracy of DANNs. All values except (†) are significant, r(52), $p<0.05$. Bold text indicates better accuracy of the same DANN evaluated on one dataset compared to the other dataset. † mark the insignificant correlation values, r(52), $p>0.05$. These results indicate that global efficiency of graphs is also related to robustness of DANNs.

Supplementary Note 6 Frameworks and hyperparameters

Frameworks and corresponding packages used in our experiments are given in Supplementary Table S4. The hyperparameters used in the training and evaluation of DANNs are given in Supplementary Table S5. For the sake of procedural consistency and comparisons of results, the set of parameters other than the those mentioned in Supplementary Table S5 are kept the same as in original experiments for relational graphs by their respective authors [2].

Supplementary Table S4 Frameworks and packages used in our codebase.

	**Package name**	**Version**
**Operating systems**	Ubuntu	20.04.3
	Windows	10
	macOS	11.6
**Programming languages**	Python	3.6.15
	Matlab	R2020a
**Deep learning framework**	Pytorch	1.4.0
	torchvision	0.5.0
**Adversarial library**	RobustBench	-
	torchattacks	3.2.1
	foolbox(optional)	3.3.1
	art(optional)	1.9.0
**Miscellaneous**	scikit-image	0.17.2
	scikit-learn	0.24.2
	scipy	1.4.1
	numpy	1.19.5
	networkx	2.3
	pyyaml	5.1.2
**Adversarial attacks** (torchattacks)	FGSM	-
	PGD	-
	CW	-
**Additive noise** (scikit-image)	Gaussian	-
	Speckle	-
	Salt & Pepper	-

Supplementary Table S5 Training & eval hyperparameters for our DANN experiments.

	CIFAR-10		CIFAR-100		TinyImageNet	ImageNet
**Hyperparam**	**MLPs**	**CNNs**	**CNNs**	**ResNet29**	**ResNet18**	**ResNet18**
Epochs	200	100	350	150	75	75
Batch size	256	1024	32	512	256	450
Base lr	0.1	0.1	0.025	0.021	0.1	0.025
lr policy	Cosine					step=[0, 25, 50, 70]
Momentum	0.9					
Weight decay	0.0005	0.01	0.0005	0.01	0.006	0.0001
Drop out	-	-	FC: p=0.1	-	Conv:p=0.2, FC:p=0.5	-
Trg iterations	5	5	5	5	1	1
Eval iterations	30	30	30	30	5	5

Supplementary Note 7 Compute resources and wall-clock times

Training time for a 5-layer MLP transformed from the WS-flex random graph on CIFAR-10 dataset is approximately 7 minutes on NVIDIA TITAN RTX GPU. Each MLP was trained five times with random seed, consuming approximately 40 minutes in training the model. On the NVIDIA TITAN RTX GPU, training of all 54 MLPs on CIFAR-10 dataset approximately took 3 days. For CIFAR-100 dataset, the 54 CNNs took approximately 5 days in training the DANNs, five times each. For Tiny ImageNet experiments on the 54 ResNet-18s, the baseline model took approximately 3 hours on TITAN RTX GPU, whereas, the longest training time for a ResNet-18 was approximately 18 hours. Total time for training 54 ResNet-18 models on Tiny ImageNet was approximately 22 days. Training the baseline model of ResNet-18 on ImageNet dataset took approximately 70 hours (3 days) on TITAN RTX GPU, the longest training time for a ResNet-18 model on ImageNet was approximately 123 hours (5 days). Total training time for 54 ResNet-18 models on ImageNet was approximately 3 months with parallel training on four GPUs. All the aforementioned training times include the inference times for FGSM, PGD, and CW adversarial attacks as well as Gaussian, Speckle, and Salt&Pepper additive noise. For tracking the experiments, visualization of results, and hyperparameter tuning, we used the Weights and Biases [3] which is a freely available performance visualization platform for machine learning tasks.

Supplementary Note 8 Sample images

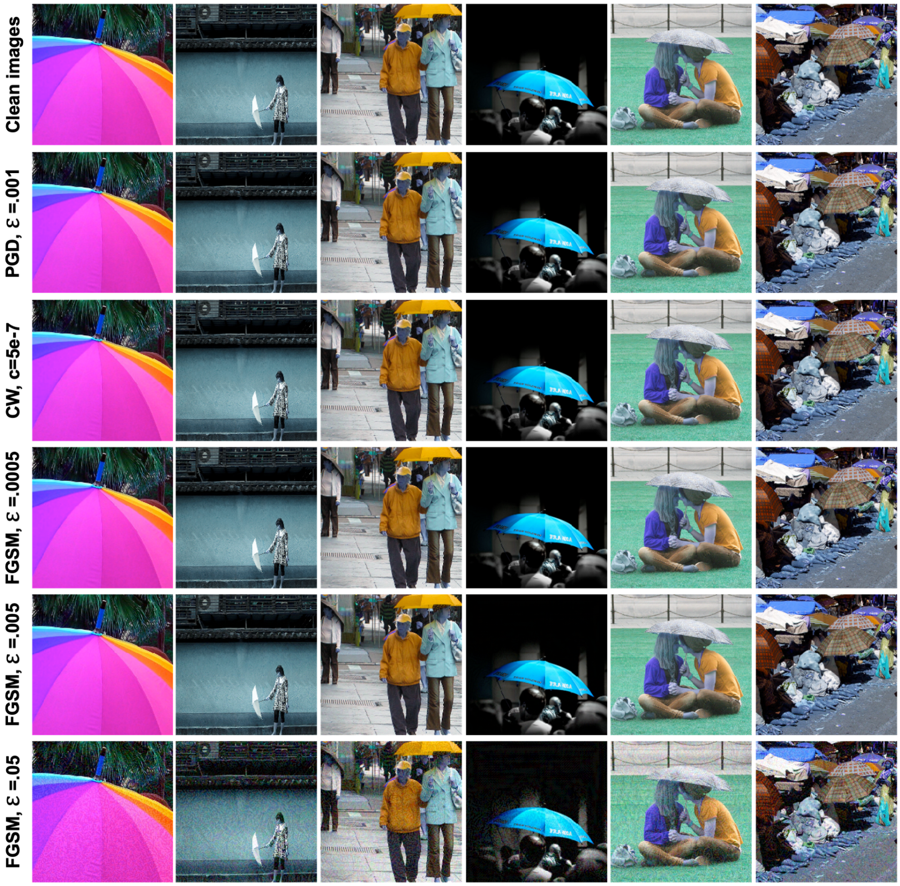

Supplementary Fig. S8 Comparison of clean images from ImageNet dataset with adversarial examples. The severity level for each adversarial attack is shown in the text of the respective row. At higher severity levels such as $FGSM(\epsilon = .05)$, the adversarial noise is noticeable.

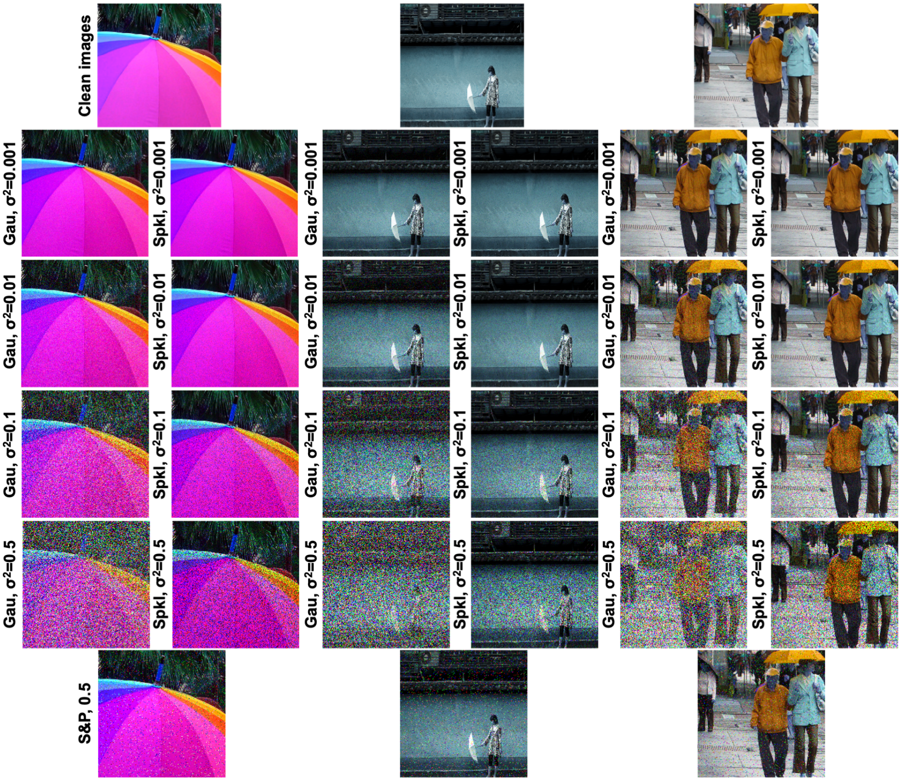

Supplementary Fig. S9 Comparison of clean images from ImageNet dataset with images having natural noise. Three noise types have been used in our experiments; Gaussian, Speckle, and Salt&Pepper. The severity level for each image is shown as a variance (σ^2) for Gaussian and Speckle noise types, and as salt vs. pepper ratio=0.5 in Salt&Pepper noise. As the severity levels increase, the images are visibly distorted.

Supplementary Note 9 Algorithm for Robust Model Selection

We propose algorithm 1 as a guideline for selecting the robust architecture of DANN for a given task without the need to undergo the train-validate-test loop for all the choices of architectures. The input parameters of the algorithm include dataset x, task t, number of nodes n, the number of DANNs to be considered α, and the binary flag about using ensemble of models. For all possible (τ) number of graphs in the given design space of n-node graphs, calculate curvature (κ_{ORC}) and entropy (H). Select α number of graphs having highest κ_{ORC} and H values, generate DANNs from these α graphs, train DANNs for the downstream task t, and evaluate under natural and malicious noise inputs. All these α DANNs have higher robustness than the rest of ($\tau - \alpha$) DANNs. Select the DANN with the highest test accuracy, or use ensemble of these α number of DANNs. Selection of parameter α is at the user's discretion as per availability of resources such as computational power and time, generally $\alpha \leq 10$.

Algorithm 1 Robust Model Selection

Input: data x, task t, nodes n, number of graphs to be selected α, ensemble
repeat
  for (τ graphs $\in$ n-node design space) **do**
    find all graphs $G_\tau(n)$
    calculate , $H(G_\tau)$, $\kappa_{OR}(G_\tau)$ (graph measures)
  end for
  for $i \leftarrow 1$ **to** α **do**
    $G_i = \max(G_\tau(H, \kappa_{OR}))$ (graphs having highest entropy, curvature)
    $[RG_\alpha] \leftarrow G_i$ (add to the list of robust graphs)
  end for
until (α **robust graphs (**RG_α**) found**)
{required number of graphs found}
repeat
  convert robust graphs to neural networks, $RG_\alpha \rightarrow NN_\alpha$
  for $j \leftarrow 1$ **to** α **do**
    train, validate $NN_j(x, t)$
    calculate test accuracy ($RobAcc(NN_j)$ with noisy, adversarial inputs
  end for
  if *ensemble* **then**
    select $Robust\ NN = avg(RobAcc(NN_j))$
  else
    select $Robust\ NN = max(RobAcc(NN_j))$
  end if
until (**Robust Neural Network found**)

Supplementary References

[1] Driss SB, Soua M, Kachouri R, Akil M. A comparison study between MLP and convolutional neural network models for character recognition. In: SPIE, editor. Real-Time Image and Video Processing 2017. vol. 10223. International Society for Optics and Photonics; 2017. p. 1022306.

[2] You J, Leskovec J, He K, Xie S. Graph Structure of Neural Networks. In: PMLR, editor. Proceedings of the 37th International Conference on Machine Learning, ICML 2020, 13-18 July 2020, Virtual Event. vol. 119 of Proceedings of Machine Learning Research; 2020. p. 10881–10891. Available from: http://proceedings.mlr.press/v119/you20b.html.

[3] Biewald L. Wandb, editor.: Experiment Tracking with Weights and Biases. wandb. Software available from wandb.com. Available from: https://www.wandb.com/.
