# Supplementary material for: Exploring robust architectures for deep artificial neural networks
Source: Commun Eng. 2022 Dec 17;1:46. doi: 10.1038/s44172-022-00043-2 (PMC10955826; doi:10.1038/s44172-022-00043-2)
Supplement: Supplementary file 3 — Description of Additional Supplementary Files [file 44172_2022_43_MOESM3_ESM.pdf]

Description of Additional Supplementary Files

File name: Supplementary Data 1

Description: Source data for graphs in the figures of main article are provided in an Excel file as Supplementary Data. Each sheet of the Excel file corresponds to the respective figure of the main article.
