# Supplementary material for: Exploring robust architectures for deep artificial neural networks
Source: Commun Eng. 2022 Dec 17;1:46. doi: 10.1038/s44172-022-00043-2 (PMC10955826; doi:10.1038/s44172-022-00043-2)
Supplement: Supplementary file 1 — Waqas_PR File [file 44172_2022_43_MOESM1_ESM.pdf]

nature portfolio

Peer Review File

Exploring Robust Architectures for Deep Artificial Neural Networks

Open Access This file is licensed under a Creative Commons Attribution 4.0 International License, which permits use, sharing, adaptation, distribution and reproduction in any medium or format, as long as you give appropriate credit to the original author(s) and the source, provide a link to the Creative Commons license, and indicate if changes were made. In the cases where the authors are anonymous, such as is the case for the reports of anonymous peer reviewers, author attribution should be to 'Anonymous Referee' followed by a clear attribution to the source work. The images or other third party material in this file are included in the article's Creative Commons license, unless indicated otherwise in a credit line to the material. If material is not included in the article's Creative Commons license and your intended use is not permitted by statutory regulation or exceeds the permitted use, you will need to obtain permission directly from the copyright holder. To view a copy of this license, visit http://creativecommons.org/licenses/by/4.0/.

Reviewers' comments:

Reviewer #1 (Remarks to the Author):

This paper studies the relationship of graph properties of deep artificial neural networks (DANNs) and their robustness performance. The authors empirically show (1) the positive correlation between graph entropy and test accuracy, especially on more complicated tasks; and (2) the increase of entropy vs robustness correlation as the task/model complexity increases. There are a few strengths:
(1) The paper is clearly written and easy to follow.
(2) The experiments have empirically shown the correlation between the graph metrics (computed pre-training) and the DANNs accuracy (only known after training). It suggests that the topological structures (i.e., presence/absence of the connections between neurons) can give us a hint on the performances of deep learning models.

However, there are a number of issues either unlear or requiring comprehensive analysis:
(1) In fig. 7, it seems like the entropy vs robustness correlation is increasing as the task or model complexity increases. However, there are only two numbers on the horizontal axis. It will be more convincing if the authors can show the results on task or model in-between those two numbers.
(2) The graph structure is fixed during training and the entropy can be computed before training. I wonder if the authors have considered some other metrics on the weighted graph after training? It will be interesting to see if different metrics on the binary and the weighted graph as well as the accuracy still have positive correlations.
(3) In Fig. 1, the author suggests that they start with building random, scale-free, or small-world networks (or graphs) that are later transformed into architectures of DANNs. The problem is generally, the DNN models consist of Dense layers (fully connected layers), but as presented in Fig. 1 (b), some neurons do not have output to the next layer. It looks like the authors designed a new topology of the DNN model. What is the performance of the new topology when compared to the vanilla DNN? Is it better than the fully connected layers?
(4) It looks like the DANNs generated from the random graphs are dropout in ML because dropout steps also don't use fully connected layers to train the model. The problem is dropouts use fully connected layers in the testing and validation processes. Did the authors use the fully connected layers as a testing model?
(5) The authors should accurately define the fragility of networks in the paper.
(6) The authors have considered many traditional graph-theoretic measures such as graph connectivity, clustering coefficient, centrality, and spectral measures (entropy). Can the authors provide some rationality and which we should use in practice? Also, I would suggest the authors to consider also more advanced network science concepts such as the newly proposed topological measures like node-based fractal dimension and node-based multi-fractal analysis (see "Deciphering the generating rules and functionalities of complex networks." Scientific reports 11, no. 1 (2021): 1-15.).
(7) The authors use ORC and entropy to quantify the robustness of graphs and claim that they are related to the robustness of graphs. However, many graph-theoretic measures are related to the robustness of graphs and can be used to quantify the robustness of the graphs. I would suggest the authors add the experiments on testing the degree of the correlation between the measures and the robustness of the graph.
(8) What are the particular architectures that can defend adversarial attacks in Fig 3?
(9) Only considered attribute noise, why is label noise not considered? Or OOD samples?
(10) Any guidance to neural network design choice? Please provide some more guidelines for practical settings.

Reviewer #2 (Remarks to the Author):

This paper proposes to use graph structure properties such as entropy and curvature as measures to

quantify the robustness of DANNs. These properties could be pre-examined before the training of the models. To testify the proposal, the authors conduct robustness analysis on the DANNs transformed from random graphs. Multilayer perceptrons (MLPs), convolutional neural networks (CNNs), and residual neural networks (ResNets) are used. It was shown that there exists positive correlation between graph structure properties and robustness of DANNs. Test accuracy against noise and adversarial attacks is used as a proxy metric to evaluate the robustness of DANNs.

Major comments:
1. In section 2.4, the Pearson product-moment correlation coefficient values were used with p values as metrics to test the correlation between graph structure properties and robustness of DANNs. To make the results more convincing, the authors could consider establishing a valid baseline. e.g. line 528 mentions that the correlation coefficient is 0.73 between entropy and accuracy. It is not clear to me how to interpret the significance of the value 0.73. Is it possible that there are other parameters other than entropy that could have stronger correlations with the test accuracy? If r = 0.73 is a good number to demonstrate the strong correlation, how should we interpret, for example, the other r values in Figure 5 which are all smaller than 0.73? The authors could analyze more systematically if the graph structure properties are more sensitive to certain types of noise and adversarial attacks, and vice versa.

The paper mentions that the recent autoML and NAS techniques could used the graph structure properties to pre-examine the robustness of their models. The authors could consider giving more proper literature review on autoML and NAS regarding how they could apply the proposed method to develop more robust DANNs.

Reviewer #3 (Remarks to the Author):

The paper presents an original analysis of the robustness of Deep Artificial Neural Networks (DANN) to adversarial perturbations. In particular, the paper focuses on the relationship between "graph-theoretic" measures of DANN, which are often studied to predict the "proficiency" of a given DANN architecture (e.g., such measures may suggest which architecture to use to maximize accuracy at test-time). However, according to the authors, such measures have not been studied (yet) in the context of adversarial perturbations. The analyses made in the paper are both theoretical and empirical: through several evaluations on well-known datasets of images, the paper elucidates the advantages

Overall, the presentation of the paper is not appropriate.
The quality of the English text is
Figures are appreciable.
The topic addressed by the manuscript is source of a large body of literature, but still an open issue and relevant to the venue.
The references are not appropriate
The contribution is (potentially?) significant.

STRENGTHS:
+ Evaluation on several datasets
+ Valuable results

WEAKNESSES
- Only image data
- Poor treatment of previous work
- Strange structure and organization

COMMENT
The paper is undoubtedly interesting, especially given the “practical” implications of its findings: by finding the correlation between “performance” and “adversarial robustness” associated to popular methods for “automatically tune” machine learning (ML) methods, it is possible to greatly enhance the deployment of ML in practice.
However, there are several criticalities that affect the paper that prevent me from recommending straight acceptance. Let me elaborate on each of them.

Only image data. The Abstract and the Introduction give the impression that the paper aims to propose “takeaways” for the general application of DANN. For example, in the Introduction, the author state (line 83) “clinical diagnosis, autonomous driving, financial decision-making, and defense.” However, the rest of the paper is based exclusively on applications of DANN for Computer Vision, i.e., where the task is recognizing “objects” in images. For example, it is not possible to determine whether the findings of the paper apply also to applications of DANNs for different tasks (e.g., cyberthreat detection [B, C], which is a domain that implicitly has to take attackers into account, and hence more aligned with the ultimate goal of the paper). This is a problem, because – as stated by the authors on line 83 – there are a plethora of applications of DANNs. To fix this issue, I recommend to either: (i) state very early on that the paper focuses just on “image” applications of DANNs (but this would decrease the impact of the paper); or (ii) do some experiments also on different datasets to showcase the generalizability of the proposed findings.

Poor treatment of previous work. This issue stems from the list of referenced works (42 in total). Most of such works are published BEFORE 2020 (only 3 are more recent). I find it rather odd that, in the last 2 years, there has been no effort in investigating the relationship between graph algorithms, adversarial robustness, and DANNs. For example, a quick search on Google Scholar returns [D, E, F] – all of which appear to tackle similar problems as the ones analyzed in this paper. At the same time, however, all past literature focuses on image data (which further strengthens my previous “weakness”). Finally, some works are reported incorrectly (e.g., “Citeseer”) or with mixed information. This “weakness” is aggravated by the lack of a “Related Work” section (or subsection) that discusses recent papers on similar subjects. Such a lack is significant: as a reviewer (but also as a reader), I am not able to fully gauge whether the main proposal of the paper is truly novel or not.

Strange structure and organization. I found the paper very difficult to read due to its organization. After a rather lengthy introduction, the second Section is entitled “Results”. There is no further introduction to the considered topic. For instance, the paper predominantly on the “topological entropy and curvature” metrics (see line 135). It is not clear “why” the focus is on just these two metrics. To a reader who is unaware of the “background content” (such as this very reviewer!) it is impossible to determine the “background context” of the paper---therefore questioning its overall contribution to the state of the art. Indeed, all such “results” are presented “as is”: as a reviewer with expertise on adversarial machine learning – but not on the specific domain considered by the paper – it is difficult to gauge whether such experiments are carried out appropriately and allow to derive the answers to the research questions tackled by the paper. Some information on this is presented only in Section 4 “Methods”: however, it is annoying as a reader to have to reach Section 4 before understanding some of the technical details of the paper. I invite the authors to restructure their paper significantly to allow even “uninformed” readers to understand the entirety of the manuscript.

Some additional issues:

- The following statement is incorrect: “The malicious or adversarial noise is imperceptible to human eye” (line 93). It is not necessary for adversarial noise to be “imperceptible to the human eye”. This is a misconception born from the Computer Vision domain where adversarial examples are usually created by minimal perturbations. In reality, a real attacker only cares about whether their attack is successful or not. See [A]

EXTERNAL REFERENCES

[A]: Carlini, Nicholas, et al. "On evaluating adversarial robustness." arXiv preprint arXiv:1902.06705 (2019).

[B]: Apruzzese, Giovanni, et al. "Modeling realistic adversarial attacks against network intrusion detection systems." ACM Digital Threats: Research and Practice (2021).

[C]: Pierazzi, Fabio, et al. "Intriguing properties of adversarial ml attacks in the problem space." 2020 IEEE symposium on security and privacy (SP). IEEE, 2020.

[D]: Zhao, Xin, et al. "Expressive 1-lipschitz neural networks for robust multiple graph learning against adversarial attacks." International Conference on Machine Learning. PMLR, 2021.

[E]: Zügner, Daniel, et al. "Adversarial attacks on graph neural networks: Perturbations and their patterns." ACM Transactions on Knowledge Discovery from Data (TKDD) 14.5 (2020): 1-31.

[F]: Jin, Wei, et al. "Adversarial attacks and defenses on graphs." ACM SIGKDD Explorations Newsletter 22.2 (2021): 19-34.

[G]: Tao, Shuchang, et al. "Adversarial Immunization for Certifiable Robustness on Graphs." Proceedings of the 14th ACM International Conference on Web Search and Data Mining. 2021.

[H]: Khoshavi, Navid, et al. "Entropy-based modeling for estimating adversarial bit-flip attack impact on binarized neural network." 2021 26th Asia and South Pacific Design Automation Conference (ASP-DAC). IEEE, 2021.

Response to Comments

Nature Communications Engineering

Title: Exploring Robust Architectures for Deep Artificial Neural Networks

Manuscript # : COMMSENG-22-0059A

The authors would like to thank the reviewers for their valuable comments and constructive suggestions. We are grateful for the opportunity to submit a revised version of the manuscript. We feel that the revision has strengthened the manuscript.

Please find below point-by-point replies to the reviewers' comments. In this document, reviewers' general comments are in **bold** text, the comments requiring our input are in blue text, and our responses are in black text. The modifications in the revised manuscript are in red text as additions and ~~deletions~~, please refer to the attached file "Revisions_for_reviewers.pdf" .

<u>Reviewer # 1</u>:

Comments to the Author:

This paper studies the relationship of graph properties of deep artificial neural networks (DANNs) and their robustness performance. The authors empirically show (1) the positive correlation between graph entropy and test accuracy, especially on more complicated tasks; and (2) the increase of entropy vs robustness correlation as the task/model complexity increases. There are a few strengths:

(1) The paper is clearly written and easy to follow.

(2) The experiments have empirically shown the correlation between the graph metrics (computed pre-training) and the DANNs accuracy (only known after training). It suggests that the topological structures (i.e., presence/absence of the connections between neurons) can give us a hint on the performances of deep learning models.

However, there are a number of issues either unclear or requiring comprehensive analysis:

1. In fig. 7, it seems like the entropy vs robustness correlation is increasing as the task or model complexity increases. However, there are only two numbers on the horizontal axis. It will be more convincing if the authors can show the results on task or model in-between those two numbers.

<u>Our Response</u>:

We thank the reviewer for insightful comments and constructive suggestions. The reviewer has rightly identified the scope and contributions of our work.

a) <u>Model Complexity Analysis</u>. To address reviewer's comment about effect of model complexity in Fig. 7, we have carried out additional experiments. Specifically, we have transformed our set of graphs to ResNet-41 architecture and trained them on CIFAR-100 task. For comparison, we tested the ResNet-41 DANNs under the same adversarial attacks and additive noise levels as already reported ResNet-29s and vanilla CNNs, as shown in *Fig. 7b* of the updated manuscript. We have also updated the manuscript to include the analysis of these additional experiments in *section 2.5*.

The statistical trends in new experiments endorse our previous findings that the significance of entropy-robustness correlation increases as the model complexity increases. This increase in correlation is directly proportional to the difference between number of parameters between the models. The entropy-robustness correlation increases significantly ($\mathrm{t}(25) = -5.85, \mathrm{p} < 0.05$) when model complexity increases from 0.5 Million parameters (in ResNet-41) to 1.3 Million (in vanilla CNNs) for the same task (CIFAR-100).

b) Task Complexity Analysis. To investigate the effect of task complexity in Fig. 7, we conducted the statistical analysis between two different models on two different tasks. We compared 8-layer CNNs evaluated on CIFAR-100 with ResNet-18s evaluated on Tiny ImageNet and reported the results in the updated Fig. 7a and associated text in *section 2.5*. We notice insignificant correlation when comparing two different models on two different tasks (i.e. $\mathrm{t}(25) = -0.95, \mathrm{p} > 0.05$) using Student's t-test. This is a justified observation because t-test compares the two related samples, whereas, our samples belong to two unrelated tasks and models. For this purpose, we used F-test to see the equality of the two unrelated populations (CNNs vs. ResNet-18s) for performance variance on separate tasks. We notice significance difference between the two populations ($F(14,14) = 2.61, p < 0.05$).

Please find below the excerpt from updated manuscript for *Fig. 7* and associated text in *section 2.5*, additions highlighted in red text. The struck-out figure and text are the deletions from the manuscript.

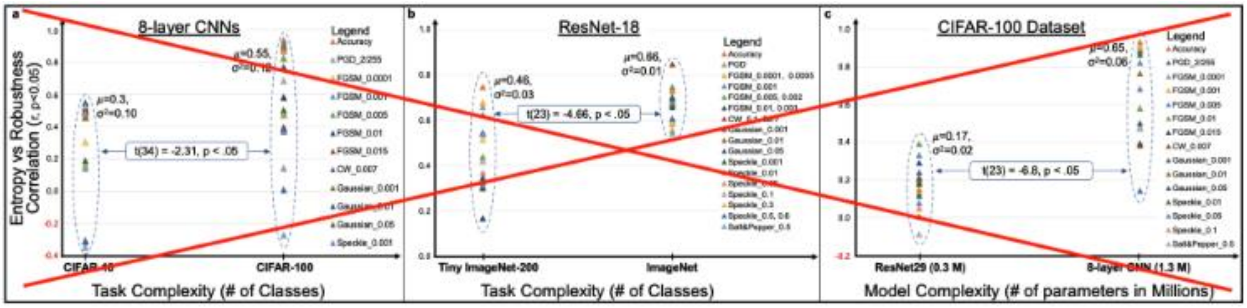

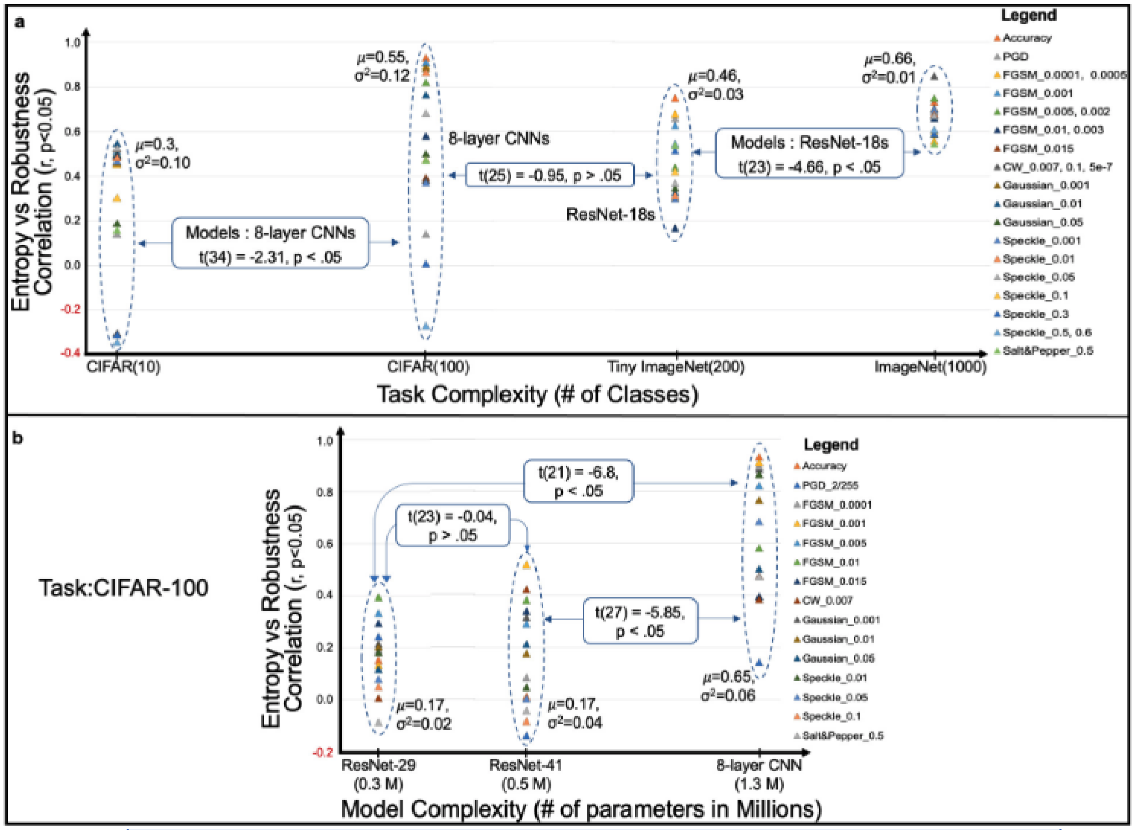

2.5 Effect of task and model complexity

using the same ResNet-18 models ($t = -4.66, n = 23, p < .05$), as shown in Fig. 7(b). Comparing two different models evaluated on separate tasks, we notice insignificant increase in the correlation values (i.e., p>0.05) as in the case of 8-layer CNN models evaluated on CIFAR-100 dataset versus ResNet-18 models evaluated on Tiny ImageNet dataset. While Student's t-test is used to compare two related samples, we used the F-test to see the equality of the two unrelated populations (CNNs vs. ResNet-18s) for performance variance on separate tasks. We observe that there is significance difference between variances of the two analyzed sets of experiments, $F(14, 14) = 2.61, p < 0.05$. In Fig. 7(c)(b), we present the effect of increasing the model complexity measured by the number of parameters against the entropy-robustness correlation. We observe that for the same CIFAR-100 dataset, as the model complexity increases from ~0.3 M parameters in ResNet-29 to ~1.3 M in CNN, the entropy-robustness correlation increases significantly ($t = -6.8, n = 23, p < .05$). Similarly, the entropy-robustness correlation increases significantly ($t = -5.85, n = 27, p < .05$) when model complexity increases from ~0.5 M parameters in ResNet-41

to ~1.3 M in CNN for CIFAR-100 task. We also note that this increase in significance is large when difference between the number of parameters between models is large. Our analysis of DANNs' robustness show that a correlation exists between graph entropy and robustness of DANNs, and this correlation has a strong dependence on the complexity of task and model.

2. The graph structure is fixed during training and the entropy can be computed before training. I wonder if the authors have considered some other metrics on the weighted graph after training? It will be interesting to see if different metrics on the binary and the weighted graph as well as the accuracy still have positive correlations.

Our Response:

We have not considered the weighted graphs of trained DANNs in our experiments. We proffer following in this regard:

a) The graph-to-DANN transformation in our experiments is reversible, i.e., we can convert the DANNs back to binary graphs using relational graph transformation [2]. The structure of the model in the graph domain remains the same. Prime consideration that stopped us from using trained DANNs as weighted graphs is that learned weights of DANNs are often devoid of scrutable structure [1]. The structure itself varies with each change in the learned weight. These compulsions must be overcome through customized mechanisms of assigning weights to the edges of graph from learned weights of DANN, as done by Casper *et al.* [1]. We believe that such manual manipulations in the graph design are not generalizable across tasks and models.

b) Moreover, the graph-to-DANN transformation is a one-to-many relationship, i.e., we can transform a graph into different architectures of DANNs such as MLP, CNN, ResNet, etc. Our motivation is that for the NAS/autoML community, generalizability of the given method without engineered interventions is key to acceptable implementation. Our mechanism enables this provision by giving the liberty to the users to select the robust DANN architecture suitable for task-at-hand.

3. In Fig. 1, the author suggests that they start with building random, scale-free, or small-world networks (or graphs) that are later transformed into architectures of DANNs. The problem is generally, the DNN models consist of Dense layers (fully connected layers), but as presented in Fig. 1 (b), some neurons do not have output to the next layer. It looks like the authors designed a new topology of the DNN model. What is the performance of the new topology when compared to the vanilla DNN? Is it better than the fully connected layers?

Our Response:

Thank you for identifying the mistake. We have updated *Fig. 1* accordingly, as shown in the snapshots from manuscript below. The DANNs in our study are traditional architectures (MLPs, CNNs, ResNets) with neuron connectivity based on random relational graphs wiring-pattern.

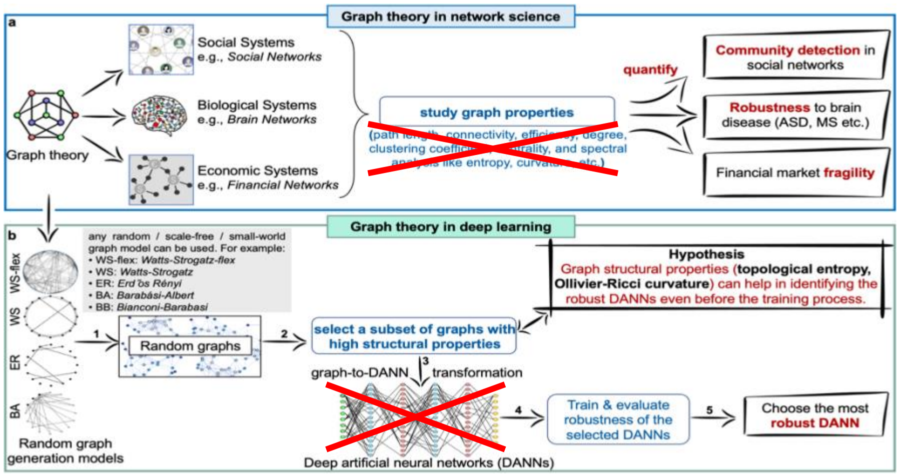

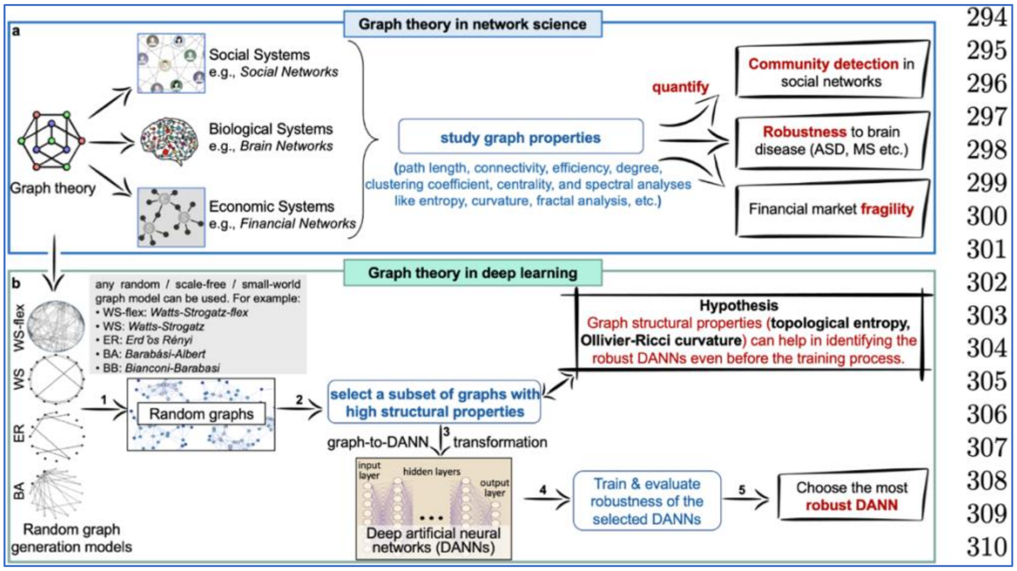

4. It looks like the DANNs generated from the random graphs are dropout in ML because dropout steps also don't use fully connected layers to train the model. The problem is dropouts use fully connected layers in the testing and validation processes. Did the authors use the fully connected layers as a testing model?

Our Response:

This is a very insightful comment by the reviewer. We want to inform the reviewer that in our experiments, the 2.313 Million graphs are down-sampled to 54 graphs of varying sparsity in the graph theory domain. These 54 graphs include a complete graph structure explicitly pointed-out in *Fig. 2a & 2e*.

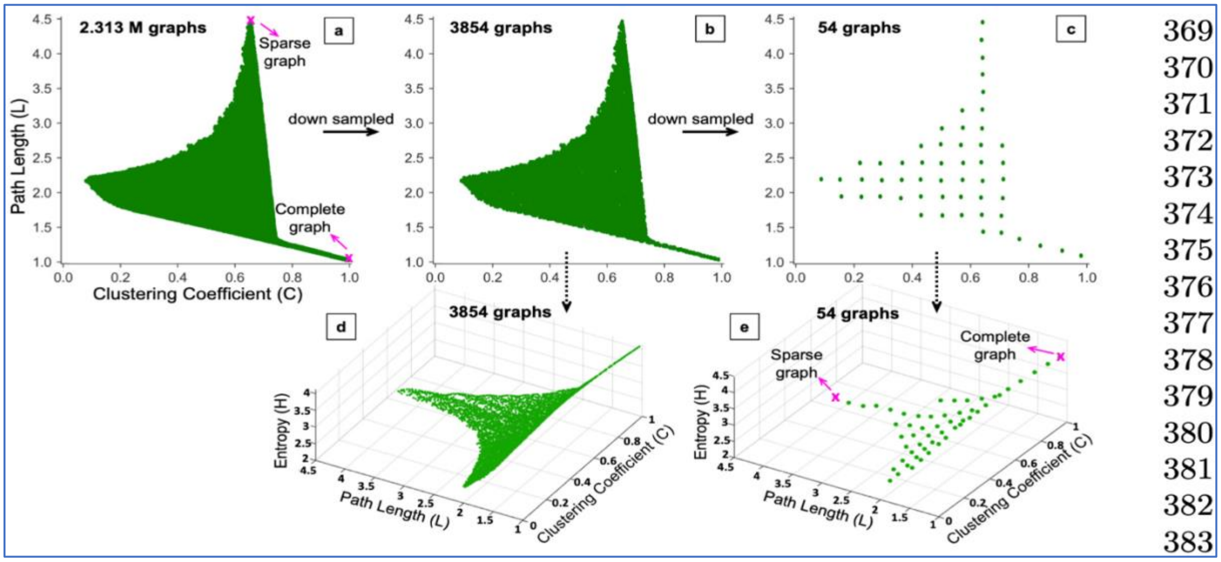

The baseline architectures of DANNs have a complete graph structure for each architecture category. For the complete graph in our experiments, $(\text{Clustering Coefficient}, \text{Path length}, \text{Entropy}) = (1.0, 1.0, 4.1)$. You *et al.* [2] have showed that the complete graph baselines are consistently out-performed by the random graphs in the deep learning domain. The resultant DANN architecture may or may not have the dropout layer. As shown in the hyperparameters *table D2* of the manuscript (copied below), we have analyzed the DANNs with and without the dropout layers.

Table D2 Training and evaluation hyperparameters for our experiments on DANNs.

	CIFAR-10		CIFAR-100		Tiny ImageNet	ImageNet
**Hyperparameter**	**MLPs**	**CNNs**	**CNNs**	**ResNet-29**	**ResNet-18**	**ResNet-18**
Epochs	200	100	350	150	75	75
Batch size	256	1024	32	512	256	450
Base lr	0.1	0.1	0.025	0.021	0.1	0.025
lr policy	Cosine					steps=[0, 25, 50, 70]
Momentum	0.9					
Weight decay	0.0005	0.01	0.0005	0.01	0.006	0.0001
Drop out	-	-	FC: p=0.1	-	Conv:p=0.2, FC:p=0.5	-
Trg iterations	5	5	5	5	1	1
Eval iterations	30	30	30	30	5	5

5. The authors should accurately define the fragility of networks in the paper.

Our Response:

Thank you for pointing out this oversight. We have included the definition and the mathematical relationship of fragility with the robustness in *Methods* section of the updated manuscript as shown in the excerpt below:

Robustness ~~(R)~~ and Fragility. We now provide the notion of robustness and fragility used in this paper. Fluctuation theorem[70] gives the concept of measuring a network's potential of returning to its "relaxed" and unperturbed state when subjected to some random perturbation. Let $p_{\gamma,\alpha}(t)$ denote the probability that under some perturbation γ at time t, the observable mean deviation of the network from its relaxed state is greater than α. ~~It is t~~The rate R at which a dynamic system returns to its original state after perturbation is given by the following function,

$$R := \lim_{t\to\infty}\left(-\frac{1}{t}\log p_{\gamma,\alpha}(t)\right). \tag{5}$$

Here, a large value of R denotes a prompt return to relaxed state after a small perturbation(γ), called the network robustness, whereas, a small R means slow return from a large perturbation(γ), called the network fragility. In the field of thermodynamics, entropy is closely related to the rate function R from large perturbations[70,71]. Fluctuation theorem [70,72] states that, given random perturbations to the network, ~~change in robustness ΔR~~ change in system entropy ΔH is positively correlated to ~~change in system entropy ΔH~~ change in robustness ΔR, and negatively correlated to change in fragility ΔF, (since $\Delta R := -\Delta F$).

$$\Delta H \times \Delta R > 0, \tag{6}$$

$$\Delta H \times \Delta F \leq 0. \tag{7}$$

6. The authors have considered many traditional graph-theoretic measures such as graph connectivity, clustering coefficient, centrality, and spectral measures (entropy). Can the authors provide some rationality and which we should use in practice? Also, I would suggest the authors to consider also more advanced network science concepts such as the newly proposed topological measures like node-based fractal dimension and node-based multi-fractal analysis (see "Deciphering the generating rules and functionalities of complex networks." Scientific reports 11, no. 1 (2021): 1-15.).

Our Response:

a) The graph-theoretic measures related to the vulnerability and robustness of the networks are generally categorized into (1) graph connectivity measures, (2) adjacency spectrum measures, and (3) Laplacian spectrum measures [3]. We have considered all three categories to study DANNs' robustness. Our analysis suggests that graph entropy and curvature are better metric for quantifying the robustness of DANNs. Our findings are based on the empirical evidence presented in our paper and these findings are reinforced by the previous endeavors in studying robustness in the Network Science (see *lines 573-580* and *bibliography items [24-28, 33]* of the revised manuscript).

b) Recently, more advanced network topological measures have been proposed. These include fractal analysis (suggested by the reviewer) [4,5], and fitness-based network efficiency [6]. We have included these works in our revised manuscript as part of the related literature. Please see *lines 177-190* of the revised manuscript for reference. However, we note that graph and node-based (multi)fractal analysis[4] quantify the importance of the edges for pruning through the measures already considered in our analysis, Ollivier–Ricci curvature (ORC) and betweenness centrality. Based on ORC and centrality ranking of edges, Xiao *et al.* [4] use Watts-Strogatz model to study the effect of edge removal on the structure of networks. Moreover, Torre *et al.* [5] study the fractality in networks using the skeleton of a complex network that is linked to betweenness centrality of the network (see equation # 2 in ref [5]). Multi-fractal analysis is essentially different from our theme of work because we do not modify the structure of the graph to study its robustness. The aim of our work is to suggest the concomitant robust architectures of DANNs barring any modifications.

c) Similarly, Ratnayake *et al.* [6] have suggested a fitness-based efficiency metric to quantify the robustness of complex networks with heterogeneous nodes. The similarity of this work with ours is that Ratnayake *et al.* [6] proposed efficiency metric which is the normalized path length used in our graph measures. However, they have considered complex networks with heterogeneous nodes. We do not consider the type of the nodes in the graph and thus our method is independent of such distinction in graph elements.

7. The authors use ORC and entropy to quantify the robustness of graphs and claim that they are related to the robustness of graphs. However, many graph-theoretic measures are related to the robustness of graphs and can be used to quantify the robustness of the graphs. I would suggest the authors add the experiments on testing the degree of the correlation between the measures and the robustness of the graph.

Our Response:

a) Many graph-theoretic measures are related to robustness of graphs. As stated in our response (a) to *comment 6* above, these measures are categorized into three types. We have also mentioned that we considered all these categories in our analysis. In *section 4.6* (*lines 1317-1328*) of the updated manuscript, we have included the interpretation of the degree of correlations between variables. As suggested, we conducted the additional experiments to test the degree of correlation of other measures, mentioned in Supplementary *Fig. B7*, with the robustness of the DANNs. We find that other than entropy and curvature, two other graph measures (*average degree*, *global efficiency*) depict strong correlations with the test accuracy. We have added these additional results in Supplementary *appendices D & E*, respectively. The correlation of average degree with robustness is not as strong as the rest of the lot, whereas, global efficiency has stronger correlations with test accuracy.

b) Efficiency between two vertices *i* and *j* is defined to be the inverse of the shortest path *d(i,j)* between them. Global efficiency of a graph is the average efficiency over all vertices $i \neq j$ [3]. Global efficiency is frequently studied graph measure in network science and it is highly dependent on the sparsity of the network [7,8]. We believe that global efficiency alone is not the appropriate measure for quantifying the robustness

of networks. It should be studied as a complimentary information on graphs, especially random and small-world graphs. As shown by Lee at al. [9], global efficiency is dependent on characteristic path length and hence cannot characterize scale-free networks such as Watts-Strogatz and its variants used in our experiments. Lee at al. [9] have also shown that volume entropy, a theoretical version of topological entropy (used in our methods) without terminal vertex, is the appropriate parameter to measure the efficiency of scale-free graphs.

c) Lastly, one of the co-authors of this manuscript has extensively studied these local and global measures on brain networks and showed through ablation studies that topological entropy and curvature provide complementary metrics for robustness assessment based on the mathematical properties such as feedback redundancy, stochastic systems, and convergence to equilibrium [10].

8. What are particular architectures that can defend adversarial attacks in Fig 3?

Our Response:

As noted in [11], despite significant amount of research on defenses against adversarial examples, it is extremely difficult that a model can withstand all forms and severity levels of attacks. In *Fig. 3*, we compare the performance of different models (MLPs, CNNs, ResNets) on image classification tasks (CIFAR-10/100, Tiny ImageNet, ImageNet). None of the presented architectures can defend against all types of adversarial attacks. However, if we look at the drop in the accuracy (on vertical axis) for all models, we notice that drop in accuracy of MLPs against adversarial attacks is persistently less than the rest of the architecture types. But the DANNs in *Fig.3* have been evaluated on different tasks. For a justifiable comparison, we trained MLPs and CNNs on the same dataset (CIFAR-10), and evaluated under similar types and severity levels of adversarial attacks. If we do a fair (apple-to-apple) comparison on the same task while ignoring the required compute and time resources, MLPs are the best in robustness compared to other model architectures such as convolutional-based models. We have reported this comparison in *section 2.3.6*, also copied below:

2.3.6 Comparison of MLPs vs. CNNs on CIFAR-10

We observed that CNNs achieve higher accuracy on the clean test data as compared to MLPs on CIFAR-10 dataset. However, under adversarial conditions (FGSM, PGD, and CW attacks), the drop in the performance of CNNs is significantly higher than MLPs as shown in panels 1 and 2 of Fig. 3. The test accuracy drop is $\sim 76\%$ for CNNs compared to $\sim 33\%$ for MLPs under PGD attack. For the CW attack, the accuracy drop for CNNs is $\sim 50\%$ compared to $\sim 34\%$ for MLPs. The same trend was observed for all severity levels of the FGSM attack. Generally, as expected CNNs outperform MLPs under clean test conditions; however, MLPs are more robust to adversarial perturbations as compared to CNNs. We argue that the observed fragility of CNNs is linked to their weight sharing and shift-invariant characteristics, which was previously noted by Zhang et al. [47].

9. Only considered attribute noise, why is label noise not considered? Or OOD samples?

Our Response:

a) Attribute Noise only: The noise can be in the attributes of the input samples (attribute noise), and it can be in the associated class label (label noise). DANNs exhibit intrinsic robustness to label noise, and the test accuracy of DANNs drop only marginally against the tens of percentage increase in the label noise [12-15]. Because of this reason, we have only considered the more hostile form of sample noise, called attribute noise.

b) Out-of-distribution Samples: OOD samples are the inputs that belong to a different distribution than training data distribution for a model. The noise caused by the day-to-day changes in input data, e.g., changes in the hardware or software configurations used for processing input data, is referred to as “natural noise” in our manuscript. Natural noise can be added to the input samples using different image processing techniques. We have added three types of natural noise to our input images, Gaussian, Speckle, and Salt & Pepper types, with varying noise levels. We have used these noisy samples as the surrogate for the OOD samples in our experiments, as done in [16].

The relevant text has been updated in the manuscript, as shown in the excerpt below:

1 Introduction

In the real world, DANNs face the challenging problem of maintaining their predictive performance in the face of uncertainties and noise in the input data[13]. The noise can be in the attributes of the input samples (attribute noise), and it can be in the associated class label (label noise). DANNs exhibit

intrinsic robustness to label noise, and the test accuracy of DANNs drop only marginally against the tens of percentage increase in the label noise[14–16]. So, the term "noise" in this work refers to the more hostile sample noise, called the attribute noise. The challenge of noise in the attributes of data is

types of noise, natural and malicious. The natural noise is related to the out-of-distribution generalization. Such noise is caused by the day-to-day changes

10. Any guidance to neural network design choice? Please provide some more guidelines for practical settings.

Our Response:

a) In the revised manuscript, we start the *Results* section with the details on design space and its relationship with the Neural Architecture Search (NAS). Our design space is spanned by clustering coefficient, path length, and entropy. We then offer the reasons for selection of our choice and cite the previous works that used them.

2.1 Graph design space

Being a sub-field of autoML, Neural Architecture Search (NAS) is the process for searching suitable architectures of neural networks for a given task[34]. Design space is a component of NAS and is composed of a set of architectures of neural networks[35]. We use two graph measures, *average path length* (L) and *clustering coefficient* (C), for exploring the graph design space. Extensively used in prior works[36–38], these measures smoothly span the whole design space of the random graphs as illustrated in Fig. 2. We generate 2.313 Million (M)

b) In *section 2.2*, we have provided details about the transformation of graphs to DANNs. We have used relational graphs as the transformation framework. Because of the flexibility and generalizability of this framework, our graphs can be translated into

multiple architecture choices for DANNs, e.g., MLPs, CNNs, ResNets, EfficientNets, etc.

2.2 From graphs to DANN architectures

We transform the downsampled 54 graphs into DANNs using the technique of relational graphs proposed by You *et al.*[9]. We transform the same 54 graphs into multiple types of DANNs including, multilayer perceptrons (MLPs), convolutional neural networks (CNNs), and residual neural networks (ResNets). Because of the flexibility and generalizability of the relational graphs, our graphs to DANNs transformation framework can be translated into diverse architectures, including MLPs, CNNs, ResNets, EfficientNets, etc. We use

c) To provide guidelines on using our method in practical settings by the NAS/autoML community, we have included an algorithm at Supplementary *appendix I*, copied below:

Appendix I Algorithm for Robust Model Selection

We propose algorithm 1 as a guideline for selecting the robust architecture of DANN for a given task without the need to undergo the train-validate-test loop for all the choices of architectures. The input parameters of the algorithm include dataset x, task t, number of nodes n, the number of DANNs to be considered α, and the binary flag about using ensemble of models. For all possible (τ) number of graphs in the given design space of n-node graphs, calculate curvature (κ_{ORC}) and entropy (H). Select α number of graphs having highest κ_{ORC} and H values, generate DANNs from these α graphs, train DANNs for the downstream task t, and evaluate under natural and malicious noise inputs. All these α DANNs have higher robustness than the rest of ($\tau - \alpha$) DANNs. Select the DANN with the highest test accuracy, or use ensemble of these α number of DANNs. Selection of parameter α is at the user's discretion as per availability of resources such as computational power and time, generally $\alpha \leq 10$.

Algorithm 1 Robust Model Selection

Input: data x, task t, nodes n, number of graphs to be selected α, ensemble
repeat
  for (τ graphs $\in$ n-node design space) **do**
    find all graphs $G_\tau(n)$
    calculate , $H(G_\tau)$, $\kappa_{OR}(G_\tau)$ (graph measures)
  end for
  for $i \leftarrow 1$ **to** α **do**
    $G_i = \max(G_\tau(H, \kappa_{OR}))$ (graphs having highest entropy, curvature)
    $[RG_\alpha] \leftarrow G_i$ (add to the list of robust graphs)
  end for
until (α **robust graphs** (RG_α) **found**)
{required number of graphs found}
repeat
  convert robust graphs to neural networks, $RG_\alpha \rightarrow NN_\alpha$
  for $j \leftarrow 1$ **to** α **do**
    train, validate $NN_j(x, t)$
    calculate test accuracy ($RobAcc(NN_j)$ with noisy, adversarial inputs
  end for
  if *ensemble* **then**
    select *Robust* $NN = avg(RobAcc(NN_j))$
  else
    select *Robust* $NN = max(RobAcc(NN_j))$
  end if
until (**Robust Neural Network found**)

Reviewer # 2:

Comments to the Author:

This paper proposes to use graph structure properties such as entropy and curvature as measures to quantify the robustness of DANNs. These properties could be pre-examined before the training of the models. To testify the proposal, the authors conduct robustness analysis on the DANNs transformed from random graphs. Multilayer perceptrons (MLPs), convolutional neural networks (CNNs), and residual neural networks (ResNets) are used. It was shown that there exists positive correlation between graph structure properties and robustness of DANNs. Test accuracy against noise and adversarial attacks is used as a proxy metric to evaluate the robustness of DANNs.

Major comments:

1. In section 2.4, the Pearson product-moment correlation coefficient values were used with p values as metrics to test the correlation between graph structure properties and robustness of DANNs. To make the results more convincing, the authors could consider establishing a valid baseline. e.g. line 528 mentions that the correlation coefficient is 0.73 between entropy and accuracy. It is not clear to me how to interpret the significance of the value 0.73.

Our Response:

We thank the reviewer for the valuable comments and insightful suggestions. The reviewer has correctly understood the proposed concept of graph structural properties such as entropy and curvature, and their application to quantify robustness of DANNs.

We agree with the reviewer about absence of the reference ranges of correlation coefficient values and corresponding interpretations. As suggested by the reviewer, we have included these reference values and interpretations[16,17] of each range viz-a-viz nature of the relationship they depict between two variables in *subsection 4.6* of *Methods* sections. Please find below the relevant excerpt of the revised manuscript, additions highlighted in red text.

4 Methods

4.6 Statistical analysis

Supplementary Fig. B7. For reference, Pearson product-moment correlation coefficient r ranges from -1 to $+1$, where larger the absolute value of r, the stronger is the relationship between variables. Specifically, the absolute values of $r = 0$ indicates no relationship, $0 < r \leq 0.3$ indicates weak relationship, $0.3 < r \leq 0.4$ indicates moderate relationship, $0.4 < r \leq 0.7$ indicates strong relationship, $r > 0.7$ indicates very strong relationship, and $r = 1$ indicates perfect relationship. We used the Student's t-test to establish that average of the correlations between entropy and robustness for two types of datasets as well as two model types are statistically different. This analysis established how entropy is related to the increase in model size and task complexity. The significance level in all these analyses is set to 95%, i.e., $p < 0.05$ indicates statistically significant values.

2. Is it possible that there are other parameters other than entropy that could have stronger correlations with the test accuracy?

<u>Our Response</u>:

a) We analyzed the correlations of many graph properties (mentioned in Supplementary *Fig. B7*) against test accuracy under adversarial attacks and noise. Other than entropy and curvature, two other graph measures depicted strong correlations with test accuracy. These measures include average degree and global efficiency. We have added these results in Supplementary *appendices D & E*, respectively.

b) The correlation of average degree with robustness is not as strong as the rest of the lot, whereas, global efficiency has stronger correlations with test accuracy. Efficiency between two vertices *i* and *j* is defined to be the inverse of the shortest path *d(i,j)* between them. Global efficiency of a graph is the average efficiency over all vertices

i≠j [18]. Global efficiency is frequently studied graph measure in network science and it is highly dependent on the sparsity of the network [19,20].

c) We believe that global efficiency alone is not the appropriate measure for quantifying the robustness of networks. It should be studied as a complimentary information on graphs, especially random and small-world graphs. As shown by Lee at al. [9], global efficiency is dependent on characteristic path length and hence cannot characterize scale-free networks such as Watts-Strogatz and its variants used in our experiments.

d) Lee at al. [9] have also shown that volume entropy, a theoretical version of topological entropy (used in our methods) without terminal vertex, is the appropriate parameter to measure the efficiency of scale-free graphs.

e) Lastly, one of the co-authors of this manuscript has extensively studied these local and global measures on brain networks and showed through ablation studies that topological entropy and curvature provide complementary metrics for robustness assessment based on the mathematical properties such as feedback redundancy, stochastic systems, and convergence to equilibrium [10].

3. If $r = 0.73$ is a good number to demonstrate the strong correlation, how should we interpret, for example, the other r values in Figure 5 which are all smaller than 0.73?

<u>Our Response</u>:

Based on the reference values for the strength of correlations and corresponding significance, we have updated the text in the manuscript to explicitly categorize the reported correlation values, as shown in the excerpt below:

> along with p values are shown on each sub-plot. There was a very strong positive correlation between the two variables, $r = 0.73$, $n = 54$, $p<0.05$ for the clean test dataset. We note similar behavior under PGD and CW attacks, that is, a strong correlation between entropy and accuracy exists, $r = 0.69$ for PGD and $r = 0.85$ for CW, $p < 0.05$ for both. Similarly, strong positive correlation trends exist for various severity levels of FGSM attack, Gaussian, speckle, and salt&pepper noise. The correlations in these experiments are all statistically significant ($p < 0.05$). In general, across all types of adversarial

trained using Tiny ImageNet and tested under various noisy conditions. We observe a strong positive correlation between entropy and predictive performance under all noise conditions, except Gaussian noise where correlation is moderate. However, there is a notable decrease in the Pearson product-

ious noisy conditions. For the CIFAR-100 experiments, we observe ~~relatively~~ very strong correlation between entropy and predictive performance except for CW ($r = 0.40$, $p < .05$) ~~and~~, PGD ($r = 0.39$, $p < .05$) adversarial attacks, and salt&pepper noise ($r = 0.47$, $p < .05$). For CIFAR-10 dataset, there is a ~~significant~~ strong correlation between entropy and predictive performance except for the PGD, CW attacks and salt&pepper noise which were not statistically significant.

4. The authors could analyze more systematically if the graph structure properties are more sensitive to certain types of noise and adversarial attacks, and vice versa.

Our Response:

Thanks for your suggestion. We have summarized our analysis of the graph structures properties sensitive to various types of noise and adversarial attacks. Please refer to the last paragraph of *section 2.4 Robustness analysis*, also copied below:

2.4 Robustness analysis

We opine that the weak correlation between graph entropy and DANNs' performance under PGD and CW attacks is due to the strong nature of PGD

and CW attacks on relatively simple classification tasks of CIFAR compared to Tiny ImageNet and ImageNet. This opinion was strengthened from the evaluation results of the CNNs on a more straightforward classification task of CIFAR-10. We observe that the correlation of entropy with the predictive performance of CNNs reduces for all categories. Moreover, the entropy's correlation with accuracy under CW attack becomes negative. Under PGD attack and salt&pepper noise, it becomes insignificant with $p>0.05$ as highlighted by the red text in respective subplots of Fig. 6.

In addition, we have updated the manuscript with an additional last paragraph in the *Results* section that summarizes the overall results and robustness analyses, as shown below:

2 Results

We summarize our results and robustness analysis here. The graph structural entropy of complex DANNs, like ResNets, is a strong indicator of their robustness against all of the analyzed additive noise and adversarial attacks on complex tasks such as 1000-class ImageNet. As we reduced the task complexity to 200-class Tiny ImageNet, the entropy-robustness relationship decreased for all categories of noise and adversarial attacks. Similarly, graph entropy of relatively simple DANNs, like 8-layer CNNs, is a strong indicator of robustness against relatively simple adversarial attacks, like FGSM, and all types of additive noise on the CIFAR-100 task. But under strong adversarial attacks, such as PGD and CW, the entropy-robustness relationship reduces for 8-layer CNNs. Similar trends were observed when testing the same 8-layer CNNs on the CIFAR-10 task.

5. The paper mentions that the recent autoML and NAS techniques could use the graph structure properties to pre-examine the robustness of their models. The authors could consider giving more proper literature review on autoML and NAS regarding how they could apply the proposed method to develop more robust DANNs.

Our Response:

a) We argue in the *Introduction* section that autoML and NAS techniques are primarily focused on test accuracy on clean input samples. We have added some recent works as references to this effect, highlighted by red box in the excerpt below:

1 Introduction

ing, and reinforcement learning[1–5]. In general, the process of the development of DANN architectures is manual, iterative and time consuming. AutoML and neural architecture search (NAS) attempt to use machine learning and search the design space of DANNs for architectures that may yield maximum test data accuracy. After the selection of a suitable DANN architecture for the given task, the optimal values of the connections (parameters or weights) are found using the training dataset and the well-known gradient descent or one of its variant algorithms[6,7]. Recently, considerable research efforts have been focused on automating the laborious task of DANN architecture design and development using techniques of autoML and NAS[8–12]. However, all such efforts are primarily focused on improving the test accuracy of the DANN on the given task.

b) We present the open avenue of exploration that can give the NAS/ autoML community an assured direction of pre-examining robustness of their models especially in the design problems where robustness of DANNs is vital. Please refer to following two blocks of excerpts from the manuscript:

cation tasks. However, the relationship between the graph-theoretic measures related to the robustness (e.g., entropy and Ollivier-Ricci curvature) of the architecture of DANNs and their performance against natural and adversarial noise has never been explored. Establishing such a relationship will allow the autoML and NAS research community to design and develop robust DANNs without training and testing these architectures.

robustness relationship assumes greater importance. The autoML/NAS design problems where robustness of DANNs is vital, our analysis can help identify

robust architectures without the need to train and test these DANNs under various noisy conditions.

c) In the *Discussion* section, we have included five latest publications and review papers on NAS/autoML. Please refer to <u>*citations 10-12, 49, 50*</u> in the revised manuscript. We have also updated the manuscript with the details on adding entropy and curvature to the existing design space of NAS as a new dimension. With this dimension included

in the procedure, the NAS/ autoML algorithms and users can directly identify robust DANNs.

d) We also included an algorithm with the details of this process in Supplementary *appendix I*. Please refer to the following excerpts from manuscript with text modifications in red color:

> Currently various autoML and NAS techniques are being developed to search for accurate model architectures for the given datasets and/or tasks[10–12,48,49]. We argue that for many mission-critical applications, the robustness of these models is equally or in some cases more important than accuracy. However, as there are currently no assured ways of estimating the robustness of DANNs in the graph design space except training and testing the candidate DANNs in the deep learning domain. We suggest that the users of autoML/NAS techniques should incorporate entropy and Ollivier-Ricci curvature information into their search framework. Using the graph representation of DANNs within the defined search space, the structure and associated topological properties of DANNs, such as entropy and curvature can be studied. Integrating these structural measures with the existing performance criterion shall enable the autoML/NAS algorithms to quantitatively and qualitatively select robust DANNs instead of exhaustively searching through all possible candidates. The users and autoML/NAS algorithms can directly identify and choose the most robust model out of all the models that meet the accuracy criteria set by the user. Such a practice would allow users or autoML/NAS algorithms to choose accurate as well as robust DANNs keeping in view the application area of the machine learning model. ~~The users and autoML/NAS algorithms can identify and choose the most robust model out of all the models that meet the accuracy criteria set by the user.~~ In Supplementary appendix I, we have given an algorithm as a guideline for selecting the robust architecture of a DANN for a given design space.

Appendix I Algorithm for Robust Model Selection

We propose algorithm 1 as a guideline for selecting the robust architecture of DANN for a given task without the need to undergo the train-validate-test loop for all the choices of architectures. The input parameters of the algorithm include dataset x, task t, number of nodes n, the number of DANNs to be considered α, and the binary flag about using ensemble of models. For all possible (τ) number of graphs in the given design space of n-node graphs, calculate curvature (κ_{ORC}) and entropy (H). Select α number of graphs having highest κ_{ORC} and H values, generate DANNs from these α graphs, train DANNs for the downstream task t, and evaluate under natural and malicious noise inputs. All these α DANNs have higher robustness than the rest of $(\tau - \alpha)$ DANNs. Select the DANN with the highest test accuracy, or use ensemble of these α number of DANNs. Selection of parameter α is at the user's discretion as per availability of resources such as computational power and time, generally $\alpha \leq 10$.

Algorithm 1 Robust Model Selection

```
Input: data x, task t, nodes n, number of graphs to be selected α, ensemble
repeat
    for (τ graphs ∈ n-node design space) do
        find all graphs G_τ(n)
        calculate , H(G_τ), κ_OR(G_τ) (graph measures)
    end for
    for i ← 1 to α do
        G_i = max(G_τ(H, κ_OR)) (graphs having highest entropy, curvature)
        [RG_α] ←G_i (add to the list of robust graphs)
    end for
until (α robust graphs (RG_α) found)
{required number of graphs found}
repeat
    convert robust graphs to neural networks, RG_α→NN_α
    for j ← 1 to α do
        train, validate NN_j(x, t)
        calculate test accuracy (RobAcc(NN_j) with noisy, adversarial inputs
    end for
    if ensemble then
        select Robust NN = avg(RobAcc(NN_j))
    else
        select Robust NN = max(RobAcc(NN_j))
    end if
until (Robust Neural Network found)
```

<u>Reviewer # 3:</u>

<u>Comments to the Author</u>:

The paper presents an original analysis of the robustness of Deep Artificial Neural Networks (DANN) to adversarial perturbations. In particular, the paper focuses on the relationship between "graph-theoretic" measures of DANN, which are often studied to predict the "proficiency" of a given DANN architecture (e.g., such measures may suggest which architecture to use to maximize accuracy at test-time). However, according to the authors, such measures have not been studied (yet) in the context of adversarial perturbations. The analyses made in the paper are both theoretical and empirical: through several evaluations on well-known datasets of images, the paper elucidates the advantages

Overall, the presentation of the paper is not appropriate.
The quality of the English text is
Figures are appreciable.
The topic addressed by the manuscript is source of a large body of literature, but still an open issue and relevant to the venue.
The references are not appropriate
The contribution is (potentially?) significant.
STRENGTHS:
+ Evaluation on several datasets
+ Valuable results
WEAKNESSES
- Only image data
- Poor treatment of previous work
- Strange structure and organization

COMMENT
The paper is undoubtedly interesting, especially given the "practical" implications of its findings: by finding the correlation between "performance" and "adversarial

robustness" associated to popular methods for "automatically tune" machine learning (ML) methods, it is possible to greatly enhance the deployment of ML in practice. However, there are several criticalities that affect the paper that prevent me from recommending straight acceptance. Let me elaborate on each of them.

1. Only image data. The Abstract and the Introduction give the impression that the paper aims to propose "takeaways" for the general application of DANN. For example, in the Introduction, the author state (line 83) "clinical diagnosis, autonomous driving, financial decision-making, and defense." However, the rest of the paper is based exclusively on applications of DANN for Computer Vision, i.e., where the task is recognizing "objects" in images. For example, it is not possible to determine whether the findings of the paper apply also to applications of DANNs for different tasks (e.g., cyberthreat detection [B, C], which is a domain that implicitly has to take attackers into account, and hence more aligned with the ultimate goal of the paper). This is a problem, because – as stated by the authors on line 83 – there are a plethora of applications of DANNs. To fix this issue, I recommend to either: (i) state very early on that the paper focuses just on "image" applications of DANNs (but this would decrease the impact of the paper); or (ii) do some experiments also on different datasets to showcase the generalizability of the proposed findings.

<u>Our Response</u>:

We thank the reviewer for the positive and constructive comments.

The reviewer is correctly interpreting the scope of our work. We have developed a new understanding of the proficiency of DANNs that relates the structural concepts borrowed from graph theory and network science to the DANNs' robustness against adversaries and noise. This novel understanding can greatly enhance the deployment of robust ML models in practical settings.

a) The reviewer has rightly identified that tasks related to image data only have been evaluated in the experiments. As suggested by the reviewer, the revised version of the manuscript highlights in greater detail the scope of the current work, its applicability across other tasks, and possible future extension where this work is applicable. Please find below the relevant excerpts from different sections of the

revised manuscript that we added to address this suggestion, additions highlighted in red text.

Abstract

The architectures of deep artificial neural networks (DANNs) are routinely studied to improve their predictive performance. However, the relationship between the architecture of a DANN and its robustness to noise and adversarial attacks is less explored, especially in computer vision applications. We investigate how the robustness of DANNs on a vision task relates to their underlying graph architectures or structures. This study: (1) starts by exploring the design space of architectures of DANNs using graph-theoretic robustness measures; (2) transforms the graphs to DANN architectures to train/validate/test on various image classification tasks; (3) explores the relationship between the robustness of trained DANNs against noise and adversarial attacks and the robustness of their underlying architectures estimated via graph-theoretic measures. We show that the topological entropy and Olivier-Ricci curvature of the underlying graphs can quantify the robustness performance of DANNs. The said relationship is stronger for complex tasks and large DANNs. Our work will allow autoML and neural architecture search community to explore design spaces of robust and accurate DANNs for tasks other than computer vision.

1 Introduction

called the attribute noise. The challenge of noise in the attributes of data is further exacerbated for mission-critical application areas, such as clinical diagnosis, autonomous driving, financial decision-making, cyberspace, and defense. Although we have used computer vision for hypothesis testing in this work, we believe that the proposed concepts are equally applicable to other fields mentioned above. Ideally, a real world deployment-ready DANN should be robust

3 Discussion

We have focused our analyses in this work on the computer vision application. However, there are a plethora of other applications and tasks where robustness quantification of DANNs is important[50–53]. A possible future direction is to extend the presented analysis to more complex applications (e.g., natural language processing, graph data, and Deep Chip industry[54–58]) and larger models (e.g., Transformers, and Vision Transformers[59,60]). Given our current analysis, we anticipate that for the larger datasets, complex tasks, and huge models, the graph robustness measures will be even more relevant and will help users/autoML/NAS algorithms find robust DANN architectures.

b) We want to highlight relevancy of our work to the papers cited by the reviewer, especially in the cyberthreat domain.

  i. Apruzzese *et al*. [21] present the capabilities and circumstances required by the attacker to carry out successful adversarial attacks. Adversarial attacks are categorized as poisoning and evasion attacks based on the phase of ML operation (training vs. inference). The target space of attacks can be feature-space or the problem space [22]. Attacker's knowledge is quantified by the white-box, gray-box, or black-box attacks. This information has been added in our revised manuscript's *Introduction* section as shown in the figure below.

1 Introduction

producing an erroneous decision[17]. The adversarial attacks can be at training time (poisoning attack) or at inference time (evasion attack)[18]. The attacks themselves can be targeted in the feature-space[17] or in the problem space[19]. The attacker can have a perfect knowledge (white-box attacks), limited knowledge (gray-box attacks), or zero knowledge (black-box attacks)[19]. Here, the knowledge $\theta = (D, X, f, w)$ is a set that may contain training data D, features X, model f and its parameters w. Black-box attacks are strict subset of white-box attacks, and white-box attacks perform better than other attacks against a DANN[20]. This means that gradient-based attacks outperform gradient-free attacks[20]. Following these facts and the Kerckhoffs' principle[21], in this work we have employed white-box attacks assuming attacker's perfect knowledge at inference time. Moreover, this work focuses on evaluating the inherent robustness of DANNs to identify the architectures that have a natural relative immunity to adversaries and insults. Mechanisms on improving the robustness of DANNs is not covered in this work.

ii. Apruzzese *et al.* [21] consider three papers as case studies[23, 24, 25].

a. [23] involves evasion attacks at inference time using classical ML algorithms including Random forest, SVM, Log Regression, and KNNs. Our experiments involve adversarial attacks as well as out-of-distribution noise samples at inference time involving the highly parameterized deep models such as MLPs, CNNs, ResNets.

b. [24] describes poisoning attack where the attackers are assumed to have power on training data. The authors use KNN-based Edge Pattern Detection (EPD) algorithm called Batch-EPD Boundary Pattern (BEBP) detection algorithm as the ML model. We have assumed highest power of the attacker in

terms of model knowledge, access to data, and features set, i.e., white-box attacks. Because white-box attacks are the strict super-sets of black-box attacks, they are superior in terms of success rate against DANNs [11].

c. [25] considers attacks at inference time against botnet detectors using Deep Q-Network (DQN) that uses deep function approximator such as CNN to represent state-action value function. Our work is directly applicable to the DQNs involving CNNs as deep function approximator because one set of our experiments involve analysis of structure of convolutional-based networks.
d. Moreover, our work can help identify robust models for the cyberthreat ML techniques that involve network traffic data in the form of images to classify malware traffic using CNNs such as [26].

Comments to the Author:

2. Poor treatment of previous work. This issue stems from the list of referenced works (42 in total). Most of such works are published BEFORE 2020 (only 3 are more recent). I find it rather odd that, in the last 2 years, there has been no effort in investigating the relationship between graph algorithms, adversarial robustness, and DANNs. For example, a quick search on Google Scholar returns [D, E, F] – all of which appear to tackle similar problems as the ones analyzed in this paper. At the same time, however, all past literature focuses on image data (which further strengthens my previous "weakness"). Finally, some works are reported incorrectly (e.g., "Citeseer") or with mixed information. This "weakness" is aggravated by the lack of a "Related Work" section (or subsection) that discusses recent papers on similar subjects. Such a lack is significant: as a reviewer (but also as a reader), I am not able to fully gauge whether the main proposal of the paper is truly novel or not.

Our Response:

a) We have removed the highlighted discrepancies in the *References* section. We had constrained ourselves to only the relevant works available in published domain because of our previous experiences with objections to long list of cited literature. Because of the format constraints, we did not explicitly include the "Related Work" (sub)section. However, as suggested by the reviewer, we have increased the literature review outlook to 75 references including 31 recent works (2020 and later).

b) The works cited by the reviewer are fundamentally different than ours because they are based on learning on the graph input data, i.e., primarily GNNs. [27] proposes a neural network, ERNN, for improving the robustness of graph learning on graph data such as MUTAG, CAIDA, and DBLP. Similarly, work in [28] targets node features of input graph data to study adversarial attacks on attributed graphs, specifically focusing on graph convolutions. [29] studies the GNNs under attacks by modifying the graph structure of input data. [30] proposes the graph adversarial immunization problem to improve the robustness of GNNs against adversarial attacks, evaluated on graph input data such as citation and social network datasets. However, our work studies the concomitant graph structures of the architecture of DANNs without considering the type of input data or the task-at-hand. [31] evaluates the resiliency of pre-trained

quantized neural networks (QNN) under hardware-driven adversarial attacks. QNNs are neural networks with extremely low precision (~1-bit) weights and activations, and they are studied from MLOps perspective. Moreover, work in [31] uses the term *entropy* under different standpoint as quoted below:

> "*We observe that the first layers are subjected to more sensitivity than the last layers. This finding highlights that considering equal importance for the parameters across the layers is inaccurate, an assumption that does not hold according to our preliminary assessment of layer importance. Our findings suggest then to consolidate traditional assumption by incorporating our preliminary finding for layer importance. We refer to this approach as the entropy-based modeling technique.*"

c) The novelty of our work is depicted by the fact that we have revisited the available literature on the topic and report that none of the published works viewed robustness of DANNs from graph structure point-of-view. Some recently published works that look at the architectures DANNs as a measure of robustness include;

   i. Huang *et al.* [32] have studied the impact of network width and depth on the robustness of adversarially trained DANNs. In our work, we do not adversarially train the DANNs to assess their robustness. Instead, we have devised a methodology of identifying the robust architectures of DANNs even before the training-validation cycle and verified this methodology empirically on a range of computer vision tasks at scale.

   ii. Parallel to our work, very recently Haifeng *et al.* [33] have studied one of our considered graph structural measures, Ollivier's Ricci curvature (ORC) to quantify the strength of structural connections of pair-wise nodes of GNNs. This is one of the extensions of our work that we have suggested in our *Discussion* section, and it shows the novelty and relevance of our work to the domain of deep learning, at large.

Comments to the Author:

3. Strange structure and organization. I found the paper very difficult to read due to its organization. After a rather lengthy introduction, the second Section is entitled “Results”. There is no further introduction to the considered topic. For instance, the paper predominantly on the “topological entropy and curvature” metrics (see line 135). It is not clear “why” the focus is on just these two metrics. To a reader who is unaware of the “background content” (such as this very reviewer!) it is impossible to determine the “background context” of the paper---therefore questioning its overall contribution to the state of the art. Indeed, all such “results” are presented “as is”: as a reviewer with expertise on adversarial machine learning – but not on the specific domain considered by the paper – it is difficult to gauge whether such experiments are carried out appropriately and allow to derive the answers to the research questions tackled by the paper.
Some information on this is presented only in Section 4 “Methods”: however, it is annoying as a reader to have to reach Section 4 before understanding some of the technical details of the paper. I invite the authors to restructure their paper significantly to allow even “uninformed” readers to understand the entirety of the manuscript.

Our Response:

a) The current format of the manuscript is pre-arranged to the journal’s format given here that requires *Methods* section to be after the *Results* and *Discussion* sections because it appears only in the online version of the accepted papers. Notwithstanding, we should have rearranged the layout for the review process.

b) Within the *Introduction* section, we have improved the draft to introduce the considered concepts gradually and logically with justifications. We start by mentioning (1) why studying underlying structure of networks is important, (2) how entropy and curvature have successfully quantified robustness in other fields, (3) what other graph measures are related to the vulnerability of networks, (4) which robustness measures are studied in this work and the reasons for our choice, and (5) what are explicit contributions of our work. Please see the excerpt from manuscript below:

1 Introduction

It has been shown with the help of Percolation theory that the architecture or structure underlying a network of any real-world system may play a key role in defining its robustness to various insults and attacks[22]. Graph-theoretic measures, such as network topological entropy and Ollivier-Ricci curvature, successfully quantify the functional robustness of various networks[23]. Examples include studying the behavior of cancer cells, analyzing the fragility of financial networks, studying the robustness of brain networks, tracking changes attributable to age and Autism Spectrum Disorder (ASD), and explaining cognitive impairment in Multiple Sclerosis (MS) patients[24–27]. Recently, the relationship between the architectures of DANNs (quantified by various graph-theoretic measures before training) and their predictive accuracy (available after training) has been established[8,9]. Various graph-theoretic measures (e.g., path length and clustering coefficient) calculated from the architectures of DANNs are quantitatively linked to their accuracy on various image classification tasks. However, the relationship between the graph-theoretic measures related to the robustness (e.g., entropy and Ollivier-Ricci curvature) of the architecture of DANNs and their performance against natural and adversarial noise has never been explored. Establishing such a relationship will allow the autoML and NAS research community to design and develop robust DANNs without training and testing these architectures.

Graph-theoretic measures that are related to the vulnerability and robustness of networks can be categorized into graph connectivity measures, adjacency spectrum measures, and Laplacian spectrum measures[28]. Based on the graph properties such as Ollivier-Ricci curvature, betweenness centrality,

and shortest path length between nodes, more advanced network measures have been recently proposed. For example, graph and node-based multifractal analysis[29,30], and fitness-based network efficiency for heterogeneous nodes[31] quantify the topology and robustness of complex networks. In this work, we study graph-theoretic properties of architectures of DANNs to quantify their test-time robustness. Specifically, we use the graph measures of topological entropy and curvature of the architecture of DANNs as robustness metrics. We have considered all three aforementioned categories of graph-robustness measures in our experiments. Our choice of reporting the curvature and entropy as the robustness measures of DANNs is based on empirical evidence presented in this paper. We make two distinct research contributions to the robustness analysis of DANNs: (1) We establish a quantitative relationship between the graph-theoretic robustness measures of entropy and curvature of DANNs (available before training) and the robustness of these DANNs to natural and adversarial noise (evaluated after training DANNs). Previous studies explored graph measures that relate to the performance of DANNs, but robustness of DANNs through graph-robustness measures has never been studied. We show that graph entropy and curvature are related to DANNs' robustness and these structural measures can identify robust architectures of DANNs even before training for the given task. (2) We show that relationship between the graph robustness measured using entropy and Ollivier-Ricci curvature and the robustness performance of DANN against noise and adversarial attacks becomes significantly stronger for complex tasks, larger datasets, and bigger

<u>Comments to the Author</u>:

4. The following statement is incorrect: “The malicious or adversarial noise is imperceptible to human eye” (line 93). It is not necessary for adversarial noise to be “imperceptible to the human eye”. This is a misconception born from the Computer Vision domain where adversarial examples are usually created by minimal perturbations. In reality, a real attacker only cares about whether their attack is successful or not. See [A]

<u>Our Response</u>:

We thank the reviewer for pointing out this mistake. As suggested, we have modified the text as shown below:

> for processing input data. The malicious or adversarial noise ~~is imperceptible to human eye and~~ is generated by an adversary for fooling the DANN into producing an erroneous decision[17]. The adversarial attacks can be at training

References

[1] Casper, Stephen, Shlomi Hod, Daniel Filan, Cody Wild, Andrew Critch, and Stuart Russell. "Graphical Clusterability and Local Specialization in Deep Neural Networks." In *ICLR 2022 Workshop on PAIR {\textasciicircum*. 2022.

[2] You, Jiaxuan, Jure Leskovec, Kaiming He, and Saining Xie. "Graph structure of neural networks." In *International Conference on Machine Learning*, pp. 10881-10891. PMLR, 2020.

[3] Freitas, Scott, Diyi Yang, Srijan Kumar, Hanghang Tong, and Duen Horng Chau. "Graph Vulnerability and Robustness: A Survey." *IEEE Transactions on Knowledge and Data Engineering* (2022).

[4] Xiao, Xiongye, Hanlong Chen, and Paul Bogdan. "Deciphering the generating rules and functionalities of complex networks." *Scientific reports* 11, no. 1 (2021): 1-15.

[5] Rendón de la Torre, Stephanie, Jaan Kalda, Robert Kitt, and Jüri Engelbrecht. "Fractal and multifractal analysis of complex networks: Estonian network of payments." *The European Physical Journal B* 90, no. 12 (2017): 1-8.

[6] Ratnayake, Prasan, Sugandima Weragoda, Janaka Wansapura, Dharshana Kasthurirathna, and Mahendra Piraveenan. "Quantifying the robustness of complex networks with heterogeneous nodes." *Mathematics* 9, no. 21 (2021): 2769.

[7] Ha, Seunggyun, Hyekyoung Lee, Yoori Choi, Hyejin Kang, Se Jin Jeon, Jong Hoon Ryu, Hee Jin Kim et al. "Maturational delay and asymmetric information flow of brain connectivity in SHR model of ADHD revealed by topological analysis of metabolic networks." *Scientific reports* 10, no. 1 (2020): 1-13.

[8] Daianu, Madelaine, Neda Jahanshad, Talia M. Nir, Arthur W. Toga, Clifford R. Jack Jr, Michael W. Weiner, and Paul M. Thompson, for the Alzheimer's Disease Neuroimaging Initiative. "Breakdown of brain connectivity between normal aging and Alzheimer's disease: a structural k-core network analysis." *Brain connectivity* 3, no. 4 (2013): 407-422.

[9] Lee, Hyekyoung, Eunkyung Kim, Seunggyun Ha, Hyejin Kang, Youngmin Huh, Youngjo Lee, Seonhee Lim, and Dong Soo Lee. "Volume entropy for modeling information flow in a brain graph." *Scientific reports* 9, no. 1 (2019): 1-13.

[10] Farooq, Hamza, Yongxin Chen, Tryphon T. Georgiou, Allen Tannenbaum, and Christophe Lenglet. "Network curvature as a hallmark of brain structural connectivity." *Nature communications* 10, no. 1 (2019): 1-11.

[11] Carlini, Nicholas, Anish Athalye, Nicolas Papernot, Wieland Brendel, Jonas Rauber, Dimitris Tsipras, Ian Goodfellow, Aleksander Madry, and Alexey Kurakin. "On evaluating adversarial robustness." *arXiv preprint arXiv:1902.06705* (2019).

[12] Flatow, David, and Daniel Penner. "On the robustness of convnets to training on noisy labels." *Technical report, Stanford University* (2017).

[13] Wang, Fei, Liren Chen, Cheng Li, Shiyao Huang, Yanjie Chen, Chen Qian, and Chen Change Loy. "The devil of face recognition is in the noise." In *Proceedings of the European Conference on Computer Vision (ECCV)*, pp. 765-780. 2018.

[14] Bar, Oshrat, Amnon Drory, and Raja Giryes. "A Spectral Perspective of DNN Robustness to Label Noise." In *International Conference on Artificial Intelligence and Statistics*, pp. 3732-3752. PMLR, 2022.

[15] Ran, Xuming, Mingkun Xu, Lingrui Mei, Qi Xu, and Quanying Liu. "Detecting out-of-distribution samples via variational auto-encoder with reliable uncertainty estimation." *Neural Networks* 145 (2022): 199-208.

[16] Ratner, Bruce. "The correlation coefficient: Its values range between+ 1/− 1, or do they?." *Journal of targeting, measurement and analysis for marketing* 17, no. 2 (2009): 139-142.

[17] Akoglu, Haldun. "User's guide to correlation coefficients." *Turkish journal of emergency medicine* 18, no. 3 (2018): 91-93.

[18] Ek, Bryan, Caitlin VerSchneider, and Darren A. Narayan. "Global efficiency of graphs." *AKCE International Journal of Graphs and Combinatorics* 12, no. 1 (2015): 1-13.

[19] Ha, Seunggyun, Hyekyoung Lee, Yoori Choi, Hyejin Kang, Se Jin Jeon, Jong Hoon Ryu, Hee Jin Kim et al. "Maturational delay and asymmetric information flow of brain connectivity in SHR model of ADHD revealed by topological analysis of metabolic networks." *Scientific reports* 10, no. 1 (2020): 1-13.

[20] Daianu, Madelaine, Neda Jahanshad, Talia M. Nir, Arthur W. Toga, Clifford R. Jack Jr, Michael W. Weiner, and Paul M. Thompson, for the Alzheimer's Disease Neuroimaging Initiative. "Breakdown of brain connectivity between normal aging and Alzheimer's disease: a structural k-core network analysis." *Brain connectivity* 3, no. 4 (2013): 407-422.

[21] Apruzzese, G., Andreolini, M., Ferretti, L., Marchetti, M. & Colajanni, M. Modeling realistic adversarial attacks against network intrusion detection systems. Digital Threats: Research and Practice (2021).

[22] Pierazzi, Fabio, Feargus Pendlebury, Jacopo Cortellazzi, and Lorenzo Cavallaro. "Intriguing properties of adversarial ml attacks in the problem space." In *2020 IEEE symposium on security and privacy (SP)*, pp. 1332-1349. IEEE, 2020.

[23] Aiken, James, and Sandra Scott-Hayward. "Investigating adversarial attacks against network intrusion detection systems in sdns." In *2019 IEEE Conference on Network Function Virtualization and Software Defined Networks (NFV-SDN)*, pp. 1-7. IEEE, 2019.

[24] Li, Pan, Qiang Liu, Wentao Zhao, Dongxu Wang, and Siqi Wang. "Chronic poisoning against machine learning based IDSs using edge pattern detection." In *2018 IEEE International Conference on Communications (ICC)*, pp. 1-7. IEEE, 2018.

[25] Wu, Di, Binxing Fang, Junnan Wang, Qixu Liu, and Xiang Cui. "Evading machine learning botnet detection models via deep reinforcement learning." In *ICC 2019-2019 IEEE International Conference on Communications (ICC)*, pp. 1-6. IEEE, 2019.

[26] Wang, Wei, Ming Zhu, Xuewen Zeng, Xiaozhou Ye, and Yiqiang Sheng. "Malware traffic classification using convolutional neural network for representation learning." In *2017 International conference on information networking (ICOIN)*, pp. 712-717. IEEE, 2017.

[27] Zhao, Xin, Zeru Zhang, Zijie Zhang, Lingfei Wu, Jiayin Jin, Yang Zhou, Ruoming Jin, Dejing Dou, and Da Yan. "Expressive 1-lipschitz neural networks for robust multiple graph learning against adversarial attacks." In *International Conference on Machine Learning*, pp. 12719-12735. PMLR, 2021.

[28] Zügner, Daniel, Oliver Borchert, Amir Akbarnejad, and Stephan Günnemann. "Adversarial attacks on graph neural networks: Perturbations and their patterns." *ACM Transactions on Knowledge Discovery from Data (TKDD)* 14, no. 5 (2020): 1-31.

[29] Jin, Wei, Yaxing Li, Han Xu, Yiqi Wang, Shuiwang Ji, Charu Aggarwal, and Jiliang Tang. "Adversarial attacks and defenses on graphs." *ACM SIGKDD Explorations Newsletter* 22, no. 2 (2021): 19-34.

[30] Tao, Shuchang, Huawei Shen, Qi Cao, Liang Hou, and Xueqi Cheng. "Adversarial immunization for certifiable robustness on graphs." In *Proceedings of the 14th ACM International Conference on Web Search and Data Mining*, pp. 698-706. 2021.

[31] Khoshavi, Navid, Saman Sargolzaei, Yu Bi, and Arman Roohi. "Entropy-based modeling for estimating adversarial bit-flip attack impact on binarized neural network." In *2021 26th Asia and South Pacific Design Automation Conference (ASP-DAC)*, pp. 493-498. IEEE, 2021.

[32] Huang, Hanxun, Yisen Wang, Sarah Erfani, Quanquan Gu, James Bailey, and Xingjun Ma. "Exploring architectural ingredients of adversarially robust deep neural networks." *Advances in Neural Information Processing Systems* 34 (2021): 5545-5559.

[33] Li, Haifeng, Jun Cao, Jiawei Zhu, Yu Liu, Qing Zhu, and Guohua Wu. "Curvature graph neural network." *Information Sciences* 592 (2022): 50-66

Reviewers' comments:

Reviewer #1 (Remarks to the Author):

Thank you for addressing reviewers' comments, please see attached file with a few remaining suggestions.

Reviewer #2 (Remarks to the Author):

The authors addressed all my questions and comments to the paper well. The quality of the paper has been much improved through the rebuttal.

Reviewer #3 (Remarks to the Author):

I thank the authors for their rebuttal. I am honestly impressed by the amount of work they put in improving the paper. I also acknowledge that my remark about the "odd structure" was misplaced for this venue (most papers on this subject have a completely different organization).

I do not have anything to add. It would have been enticing if the paper also considered a different domain than Computer Vision; however, at least the paper is transparent in what it does. Yet, I strongly invite the authors to explore this research direction instead of leaving it as a mere "future work".

Response to Comments
Nature Communications Engineering

Title: Exploring Robust Architectures for Deep Artificial Neural Networks Manuscript # : COMMSENG-22-0059A

Legend:
Blue – reviewer comment
Red – author response

Reviewer # 1:

1. [OK] "… However, there are only two numbers on the horizontal axis. It will be more convincing if the authors can show the results on task or model in- between those two numbers."
    - Additional experiments were carried out. Fig. 7 updated. "The statistical trends in new experiments endorse our previous findings that the significance of entropy-robustness correlation increases as the model complexity increases."
2. [Need clarification] "I wonder if the authors have considered some **other metrics** on the weighted graph after training?"
    - This statement was not fully addressed with regards to other metrics.
3. [OK] "The problem is generally, the DNN models consist of Dense layers (fully connected layers), but as presented in Fig. 1 (b), some neurons do not have output to the next layer."
    - Figure 1 is updated to show that the "DANNs in our study are traditional architectures."
4. [OK] "Did the authors use the fully connected layers as a testing model?"
    - "These 54 graphs include a complete graph structure explicitly pointed-out in *Fig. 2a & 2e*."
    In table D2 "We have analyzed the DANNs with and without the dropout layers"
5. [OK] "The authors should accurately define the fragility of networks in the paper."
    - "We have included the definition and the mathematical relationship of fragility with the robustness in *Methods* section."
6. [OK] "The authors have considered many traditional graph-theoretic measures such as graph connectivity, clustering coefficient, centrality, and spectral measures (entropy). Can the authors provide some rationality and which we should use in practice?"
    - "The graph-theoretic measures related to the vulnerability and robustness of the networks are generally categorized into (1) graph connectivity measures, (2) adjacency spectrum measures, and (3) Laplacian spectrum measures [3]. …"

    [OK] "I would suggest the authors to consider also more advanced network science concepts such as the newly proposed topological measures like node-based fractal dimension and node-based multi-fractal analysis"
    - [177-190] "We have included these works in our revised manuscript as part of the related literature." "However, we note that graph and node-based (multi)fractal analysis[4] quantify the importance of the edges for pruning through the measures already considered in our analysis, Ollivier–Ricci curvature (ORC) and betweenness centrality."
    - "Multi-fractal analysis is essentially different from our theme of work because we do not modify the structure of the graph to study its robustness."
    - "Ratnayake *et al.* [6] have suggested a fitness-based efficiency metric to quantify the robustness of complex networks with heterogeneous node. … We do not consider the type of the nodes in the graph and thus our method is independent of such distinction"
7. [OK] "However, many graph-theoretic measures are related to the robustness of graphs and can be used to quantify the robustness of the graphs. I would suggest the authors add the experiments on testing the degree of the correlation between the measures and the robustness of the graph."
    - Related and addressed in response (a) to comment 6 that the measures are categorized into three types and these have been considered in the analysis.
    - "As suggested, we conducted the additional experiments to test the degree of correlation of other measures, mentioned in Supplementary *Fig. B7*, with the robustness of the DANNs."
8. [OK] "What are particular architectures that can defend adversarial attacks in Fig 3?

- "If we do a fair (apple-to-apple) comparison on the same task while ignoring the required compute and time resources, MLPs are the best in robustness compared to other model architectures such as convolutional-based models. We have reported this comparison in *section 2.3.6*"

9. [OK] "Only considered attribute noise, why is label noise not considered? Or OOD samples?"
   - "DANNs exhibit intrinsic robustness to label noise, and the test accuracy of DANNs drop only marginally against the tens of percentage increase in the label noise [12-15]. Because of this reason, we have only considered the more hostile form of sample noise, called attribute noise."
   - For OOD samples, these are "referred to as "natural noise" in our manuscript. … We have added three types of natural noise to our input images, Gaussian, Speckle, and Salt & Pepper types, with varying noise levels."
10. [OK] "Any guidance to neural network design choice? Please provide some more guidelines for practical settings."
   - "To provide guidelines on using our method in practical settings by the NAS/autoML community, we have included an algorithm at Supplementary *appendix I*"

Reviewer # 2

1. [OK] "To make the results more convincing, the authors could consider establishing a valid baseline. e.g. line 528 mentions that the correlation coefficient is 0.73 between entropy and accuracy. It is not clear to me how to interpret the significance of the value 0.73."
   - [1317-1334] "We have included these reference values and interpretations[16,17] of each range viz-a-viz nature of the relationship they depict between two variables in *subsection 4.6* of *Methods* sections."
2. [OK] "Is it possible that there are other parameters other than entropy that could have stronger correlations with the test accuracy?"
   - Other graph measures are presented in appendices D & E. "two other graph measures [average degree and global efficiency] depicted strong correlations with test accuracy."
3. [Could be improved] "If r = 0.73 is a good number to demonstrate the strong correlation, how should we interpret, for example, the other r values in Figure 5 which are all smaller than 0.73?"
   - Any guidelines on how to categorize the correlation based on the numerical *r*-value.
   - [620-632] Text updated "in the manuscript to explicitly categorize the reported correlation values."
4. [OK] "The authors could analyze more systematically if the graph structure properties are more sensitive to certain types of noise and adversarial attacks, and vice versa."
   - Addressed in §2.4. Added paragraph in the results section [837-856]. "We have summarized our analysis of the graph structures properties sensitive to various types of noise and adversarial attacks."
5. [OK] "The authors could consider giving more proper literature review on autoML and NAS regarding how they could apply the proposed method to develop more robust DANNs."
   - This is in multiple sections of the paper. In the Intro [65-84] "that autoML and NAS techniques are primarily focused on test accuracy on clean input samples." "In the *Discussion* section, we have included five latest publications and review papers on NAS/autoML. Please refer to *citations 10-12, 49, 50* in the revised manuscript."

Reviewer # 3

1. [Partially addressed] To fix this issue, I recommend to either: (i) state very early on that the paper focuses just on "image" applications of DANNs (but this would decrease the impact of the paper); or (ii) do some experiments also on different datasets to showcase the generalizability of the proposed findings."
   - Abstract, intro, and discussion updated to highlight "in greater detail the scope of the current work, its applicability across other tasks, and possible future extension where this work is applicable."
   - However, no experiments on different non-image datasets.
2. [OK] "This "weakness" is aggravated by the lack of a "Related Work" section (or subsection) that discusses recent papers on similar subjects."

- “we have increased the literature review outlook to 75 references including 31 recent works (2020 and later).” “The novelty of our work is depicted by the fact that we have revisited the available literature on the topic and report that none of the published works viewed robustness of DANNs from graph structure point-of-view.”

[OK] “Finally, some works are reported incorrectly (e.g., “Citeseer”) or with mixed information.”

- “We have removed the highlighted discrepancies in the *References* section.”

3. [OK, but subjective] “Strange structure and organization. I found the paper very difficult to read due to its organization.”
    - Authors followed the journal’s format and presented their reasoning based on the organization.
4. [OK] “The following statement is incorrect: “The malicious or adversarial noise is imperceptible to human eye” (line 93).”
    - Mistake corrected in [115-118].

In addition, the authors mentioned at line 354 that "We transform the downsampled 54 graphs into DANNs using the technique of relational graphs". The reviewer wonders where the 54 graphs come from and if they are representative. Also, why do you downsample the graphs? Why not just use the existing graphs? How can you make sure that the downsampled graphs preserve the same structures as the original graphs?

<u>Response to Comments</u>

<u>Nature Communications Engineering</u>

Title: Exploring Robust Architectures for Deep Artificial Neural Networks

Manuscript # : COMMSENG-22-0059A

The authors thank all the reviewers for their valuable feedback and for helping us improve this manuscript. We truly feel that the suggested revisions have broadened the outreach of the central idea in our work as well as improved its readability.

Please find below point-by-point replies to the reviewers' comments. There are no changes to the manuscript in response to the observations mentioned in this document.

Reviewer # 1:

1. The graph structure is fixed during training and the entropy can be computed before training. I wonder if the authors have considered some **other metrics** on the weighted graph after training? It will be interesting to see if different metrics on the binary and the weighted graph as well as the accuracy still have positive correlations.

Our Response:

Premise: We thank the reviewer for their deliberation on this point. We have tried to explain our response with the help of Fig.1 below.

Consider the example of a *Complete Graph* with binary edges, as shown in Fig. 1 below. We study the graph structural properties of this *Complete Graph* using various metrics discussed in Section 4.2 and Fig. B7 of the manuscript. Using the *Relational Graph* transformation given by Equation 10 of the manuscript, we transform this complete graph into an architecture of a DANN. Because of the generalizability of the used transformation, the same graph can be used to instantiate multiple architectures of DANNs such as MLP, multiple-layered CNN, ResNet family, EfficientNet family, etc. In our experiments, we transformed the single graph into many different neural networks, including 5-layer MLP, 8-layer CNN, ResNet-18, ResNet-29, and ResNet-41 (please refer to lines 1133–1155 of the manuscript). After training the DANNs on a given task, we analyzed the performance of DANNs viz-a-viz their binary graph-structural properties. The trained DANNs can be thought of as weighted graphs and/or binary graphs. The edge labels between two nodes (neurons) of the weighted graphs depict the connection weights, whereas binary graphs have edge labels 1 (connection) or 0 (no connection) between nodes.

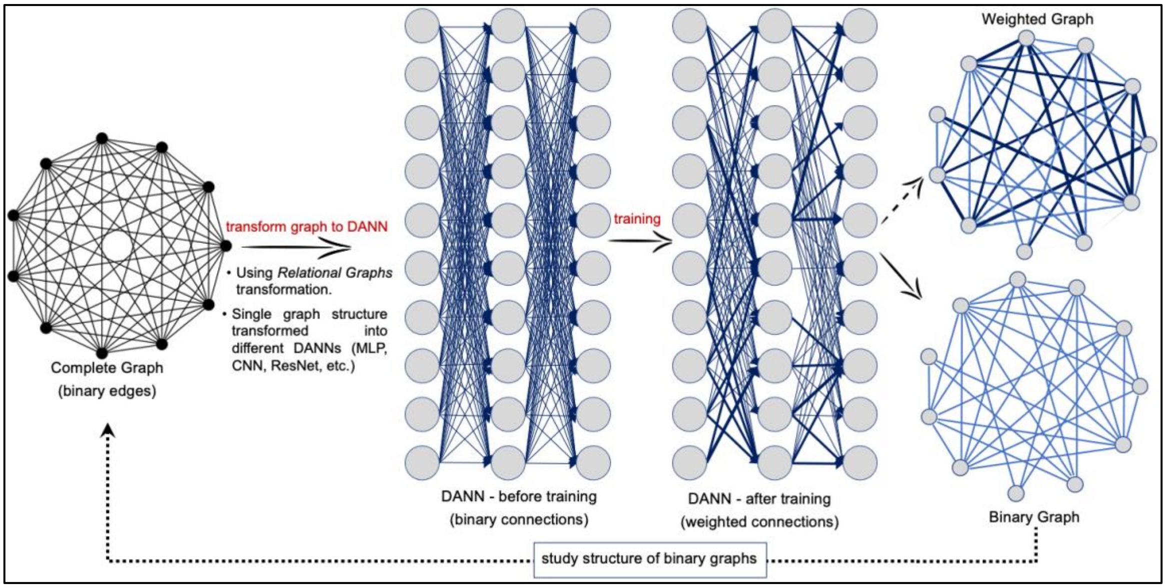

Figure 1: Binary graphs vs. weighted graphs

4.3 From graphs to DANNs

at each node through an aggregation function $F(.)$. The i-th message exchange round between nodes v and u can be expressed as,

$$\mathbf{x}_v^{(i+1)} = F^{(i)}(\{f_v^{(i)}(\mathbf{x}_u^{(i)}), \forall u \in N(v)\}). \quad (10)$$

You *et al.* have shown that Equation (10) is the general definition of message exchange that can be used to instantiate any neural architecture[9]. We generate MLP, CNN, ResNet-18, and ResNet-29 for each of the 54 random graphs generated from the WS-flex generator.

The same 54 WS-flex random graphs were transformed into a total of 216 DANNs having 54 neural networks in each of the four categories (MLP, CNN, ResNet-18, and ResNet-29). MLPs were trained on CIFAR-10 dataset,

Argument: We have considered multiple metrics to study the graph structure of **unweighted (binary) graphs only**. Moreover, we have studied these binary graphs from a graph-theoretic perspective **before training** them in the deep learning domain. We have not analyzed the weighted graphs of trained DANNs in our experiments because of the reasons mentioned below:

a) The underlying (binary) structure of the before-training DANN model in the graph domain remains the same irrespective of whether we use graph-to-DANN transformation or DANN-to-graph transformation of the relational graphs [1]. This consistent binary graph structure helped us study and compare the DANNs' robustness through the prism of Network Science and Graph Theory.

b) The weighted (graph) structure of DANN changes with every change in the DANN's weights. The DANN's weights are sensitive to input data, training epochs, learning rate, loss function, etc. Moreover, the learned weights of DANNs often lack comprehendible structure, as recently noted by Casper *et al.* [2]. Researchers resort to various customized mechanisms for assigning weights to the edges of the graph from learned weights of DANN, as done by Casper *et al.* [1]. These manual customizations in the graph design are not generalizable across tasks and models.

For the NAS/autoML community, the generalizability of the given method without engineered interventions is important for an acceptable adoption.

c) For the binary graphs, we have analyzed the robustness of DANNs using metrics such as topological entropy, Olivier-Ricci curvature, clustering coefficient, path length, average degree, betweenness centrality, efficiency, and eigenvector centrality. Our findings show that structural properties such as entropy and curvature significantly correlate to the robustness of the DANNs across various tasks and models.

d) Although the structure of the post-training weighted graphs can be analyzed using the metrics mentioned above, such analysis would not be generalizable across varying tasks and models. Even for the same task and model architecture, the weighted graph structure of the DANN would change for each change in the model's weights.

Reviewer # 3:

In addition, the authors mentioned at line 354 that "We transform the downsampled 54 graphs into DANNs using the technique of relational graphs". The reviewer wonders where the 54 graphs come from and if they are representative. Also, why do you downsample the graphs? Why not just use the existing graphs? How can you make sure that the downsampled graphs preserve the same structures as the original graphs?

Our Response:

We generated 2.313 Million candidate random graphs from the WS-flex generator, as shown in the Fig.2a of the manuscript (also pasted below). Our goal was to analyze the given design space for performance and robustness analysis of DANNs.

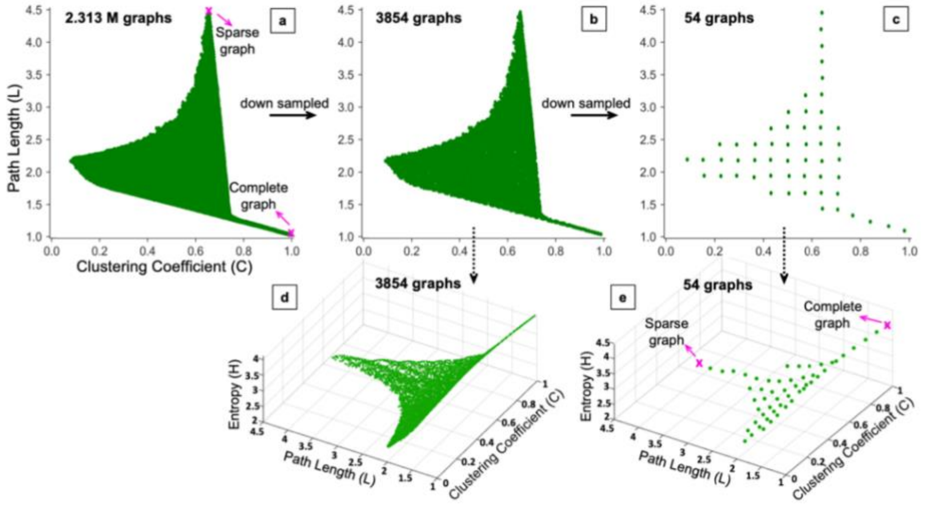

Fig. 2 The graph design space for generating random graphs. **(a),(b),(c):** show 2.313 M candidate graphs from WS-Flex generator downsampled to 3854 and 54 graphs. **(d)** and **(e)** show 3854 and 54 graphs in a 3-D space spanned by clustering coefficient (C), path length (L), and entropy (H). Samples of the complete and sparse graphs are identified in (a) and (e).

Why downsample? Transforming every candidate graph (~2 Million) into different DANNs architectures (MLP, CNN, ResNets, etc.) for training on different tasks (CIFAR-10/100, Tiny/ImageNet) was computationally challenging and very expensive. So, we had to downsize our set of candidate graphs to a reasonable number of graphs we could use to instantiate, train, and test the DANNs' robustness.

Where the 54 graphs come from and if they are representative? As mentioned on lines 324-329 of the manuscript (also pasted below), we reduce the number of graphs (downsample) from 2.313 Million to 3,854 and then further to 54 graphs through coarser bins in such a way that each of the reduced number of graphs are representative of all the graphs lying within that bin. As can be visualized in Figs 2b and 2c of the manuscript (pasted above) that the downsampled graphs consistently span the entire design space

as of the original 2.313 Million graphs. Hence, the 54 graphs are representative of the entire design space.

2.1 Graph design space

Being a sub-field of autoML, Neural Architecture Search (NAS) is the process for searching suitable architectures of neural networks for a given task[35]. Design space is a component of NAS and is composed of a set of architectures of neural networks[36]. We use two graph measures, *average path length* (L) and *clustering coefficient* (C), for exploring the graph design space. Extensively used in prior works[37–39], these measures smoothly span the whole design space of the random graphs as illustrated in Fig. 2. We generate 2.313 Million (M) candidate random graphs using Watts-Strogatz flex (WS-flex) graph generator for a range of C and L values as illustrated in Fig. 2(a). We chose WS-flex because its graphs are superset of graphs generated by three classical

methods including, Watts-Strogatz (WS), Erdős Rényi (ER), and Barabási-Albert (BA)[37,40,41]. We downsample 2.313 M candidate WS-flex graphs into coarser bins of 3854 and 54 graphs (Fig. 2(b)&2(c)), where each bin has at least one representative graph. We visualize our candidate graphs using their average path length(L), clustering coefficient (C), and entropy (H). Entropy is a graph-theoretic measure for robustness and we visualize our design space spanned by (C, L, H), as depicted in Fig. 2(d)&2(e). Fig. 2(a)&2(e) also depict

Why not just use the existing graphs? We used the existing graphs but did not use all 2.313 Million to transform them into DANNs for further analysis.

How can you make sure that the downsampled graphs preserve the same structures as the original graphs? As mentioned earlier, we did not change the structure of the graphs selected for robustness analysis.

References

[1] You, Jiaxuan, Jure Leskovec, Kaiming He, and Saining Xie. "Graph structure of neural networks." In *International Conference on Machine Learning*, pp. 10881-10891. PMLR, 2020.

[2] Casper, Stephen, Shlomi Hod, Daniel Filan, Cody Wild, Andrew Critch, and Stuart Russell. "Graphical Clusterability and Local Specialization in Deep Neural Networks." In *ICLR 2022 Workshop on PAIR {\textasciicircum*. 2022.

REVIEWERS' COMMENTS:

Reviewer #1 (Remarks to the Author):

In my opinion, the authors have addressed well my earlier comments or suggestions. I do see that I had similar questions as some of the other reviewers in terms of the nature of the graphs and how they change, but to be fair probably what I would ask will lead to more papers and that may be beyond the scope. Overall, I think the work can be published and it will spark numerous investigations.
